# Skeletal muscle status and survival among patients with advanced biliary tract cancer

**DOI:** 10.1007/s10147-023-02466-z

**Published:** 2024-02-06

**Authors:** Shinya Takaoka, Tsuyoshi Hamada, Naminatsu Takahara, Kei Saito, Go Endo, Ryunosuke Hakuta, Kota Ishida, Kazunaga Ishigaki, Sachiko Kanai, Kohei Kurihara, Hiroki Oyama, Tomotaka Saito, Tatsuya Sato, Tatsunori Suzuki, Yukari Suzuki, Shuichi Tange, Yurie Tokito, Ryosuke Tateishi, Yousuke Nakai, Mitsuhiro Fujishiro

**Affiliations:** 1 Department of Gastroenterology, Graduate School of Medicine, The University of Tokyo, Tokyo, Japan; 2 Department of Hepato-Biliary-Pancreatic Medicine, The Cancer Institute Hospital, Japanese Foundation for Cancer Research, Tokyo, Japan; 3 Department of Endoscopy and Endoscopic Surgery, The University of Tokyo Hospital, 7-3-1 Hongo, Bunkyo City, Tokyo, 113-8655 Japan

**Keywords:** Body composition, Chemotherapy, Cholangiocarcinoma, Cohort studies, Sarcopenia, Survival analysis

## Abstract

**Background:**

Studies have demonstrated a prognostic role of sarcopenia (*i.e.*, loss of skeletal muscle volume and functionality) in patients with various cancer types. In patients with biliary tract cancer, the quantity and quality of skeletal muscles and their serial changes have not been fully investigated in relation to survival outcomes.

**Methods:**

We identified 386 patients with unresectable or recurrent biliary tract cancer and calculated skeletal muscle index (SMI) and skeletal muscle density (SMD) to estimate muscular quantity and quality, respectively, based on computed tomography images. Using the Cox regression model with adjustment for potential confounders, we calculated hazard ratios (HRs) and 95% confidence intervals (CIs) for progression-free survival (PFS) and overall survival (OS) according to skeletal muscle status and its serial change.

**Results:**

Compared to patients without sarcopenia, patients with sarcopenia were associated with shorter PFS (multivariable HR, 1.60; 95% CI, 1.15–2.22; *P* = 0.005), but not with OS (*P* = 0.027) at the adjusted α level of 0.013. SMD at baseline was associated with OS (multivariable HR comparing the extreme quartiles, 1.52; 95% CI, 1.07–2.14; *P*_trend_ = 0.012), but not with PFS (*P*_trend_ = 0.13). A reduction in SMI rather than that in SMD was associated with OS. Progressive disease was a risk factor for reductions in SMI and SMD.

**Conclusions:**

Skeletal muscle quantity and quality and their serial changes were associated with survival outcomes in patients with advanced biliary tract cancer. Our data highlight the importance of designing nutritional and physical interventions for improvements in skeletal muscle status.

**Supplementary Information:**

The online version contains supplementary material available at 10.1007/s10147-023-02466-z.

## Introduction

The concept of sarcopenia, which is defined as an age-related loss of skeletal muscle volume and functionality [1, 2], has attracted attention of clinical oncologists with accumulating evidence for the remarkable role of sarcopenia in prognostication of cancer patients. Clinical studies suggest that sarcopenia may be correlated with risks of surgical complications [3, 4] and postoperative recurrence [5, 6] as well as response to and tolerance for chemotherapeutic agents [7, 8], potentially resulting in unfavorable survival outcomes of patients with various types of malignancies [9]. Sarcopenia at cancer diagnosis may serve as a surrogate for various cancer-related factors with negative impacts on survival times, including impaired nutritional status, unfavorable metabolic alterations, and sustained systemic inflammation [10]. Further research suggests that clinical outcomes of cancer patients may depend on postdiagnostic alterations in skeletal muscle status due to the invasiveness of surgery [11, 12] or the toxicity of chemotherapy [13–15]. A better understanding of the pathogenesis of skeletal muscle alterations in the context of postdiagnostic clinical course of cancer patients would help to develop and implement new management strategies of the patients [16].

In patients with biliary tract cancer, the quantity and quality of skeletal muscles have not been fully investigated in relation to survival outcomes. Inconsistent results from the prior studies have been attributable to the small sample sizes due to the relative rarity of the disease [17, 18]. In addition, survival associations and risk factors of deterioration of skeletal muscle status remain to be elucidated. Biliary tract cancer is a collection of neoplasms arising from the biliary epithelium in intrahepatic and extrahepatic bile ducts and gallbladder, which may represent heterogeneous biological behaviors [19]. Therefore, a large sample size is required to evaluate prognostic factors in a population with this malignancy overall. Considering suggestive evidence linking skeletal muscle status to the effectiveness of chemotherapy for biliary tract cancer [20, 21] as well as ample evidence on the survival associations of sarcopenia in various malignancies, we hypothesized that skeletal muscle status might be associated with survival outcomes of patients with biliary tract cancer.

To test our hypothesis, we utilized a clinical cohort of patients with biliary tract cancer and examined the associations of skeletal muscle volume and density with progression-free and overall survival times of patients with unresectable or recurrent biliary tract cancer. In addition, we examined serial alterations of skeletal muscle status in relation to survival outcomes and risk factors for unfavorable muscular alterations. We utilized a specific analytical software for images of computed tomography (CT) and measured the area and density of skeletal muscles to estimate their quantity [22] and quality [23], respectively.

## Patients and methods

### Study population

**Fig. 1:**
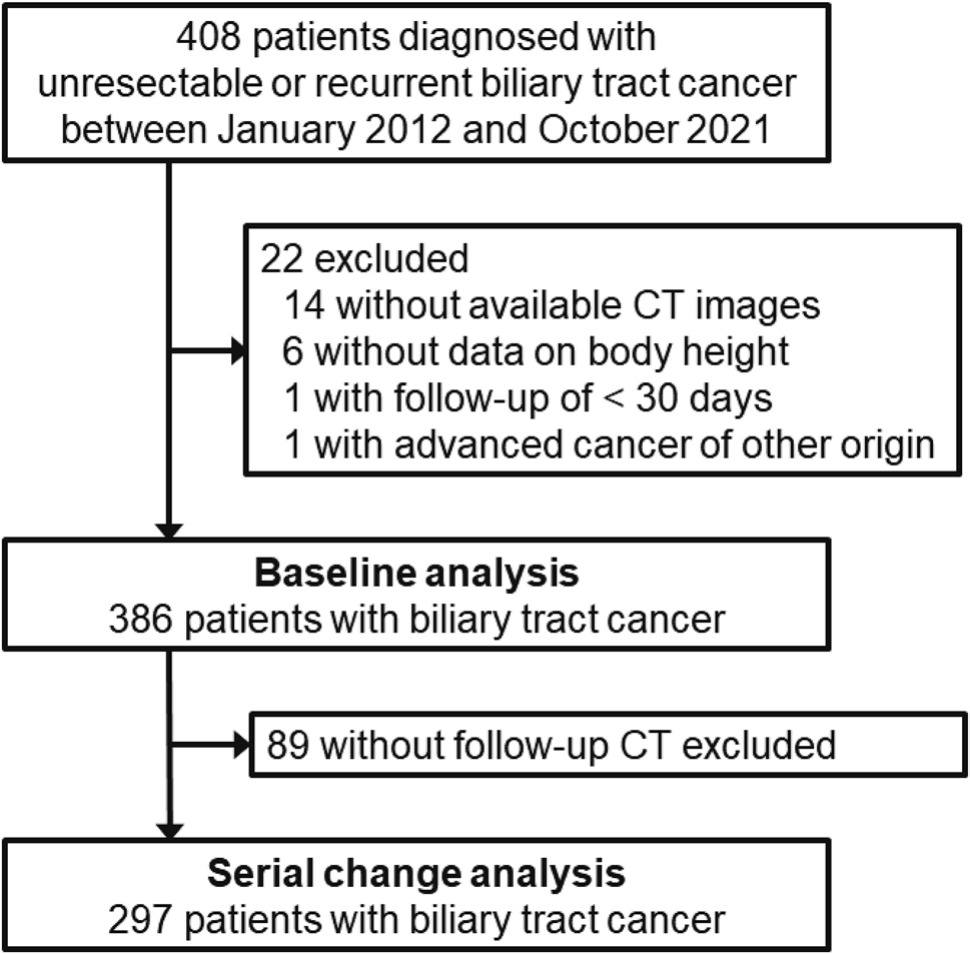
Flow diagram of selection of patients with advanced biliary tract cancer for the current study

Using our prospectively maintained database, we identified consecutive patients who were diagnosed with biliary tract cancer at The University of Tokyo Hospital (Tokyo, Japan) between Jan 2012 and Oct 2021 (Fig. 1). We excluded patients without available CT images for the analysis, patients with concomitant advanced cancer of other origin, and patients with follow-up time of < 30 days. In analyses of progression-free survival (PFS), we additionally excluded patients who received radiation or best supportive care. The patients were followed until death or the end of follow-up (Sep 30, 2022), whichever came first.

This retrospective cohort study was conducted according to the guideline in the Helsinki Declaration and was approved by the ethics committee of The University of Tokyo (Tokyo, Japan; approval number, #2058). Informed consent was obtained from the participants on an opt-out basis given the non-invasive nature of the study.

### Computed tomography (CT)-based assessment of skeletal muscle status

**Fig. 2:**
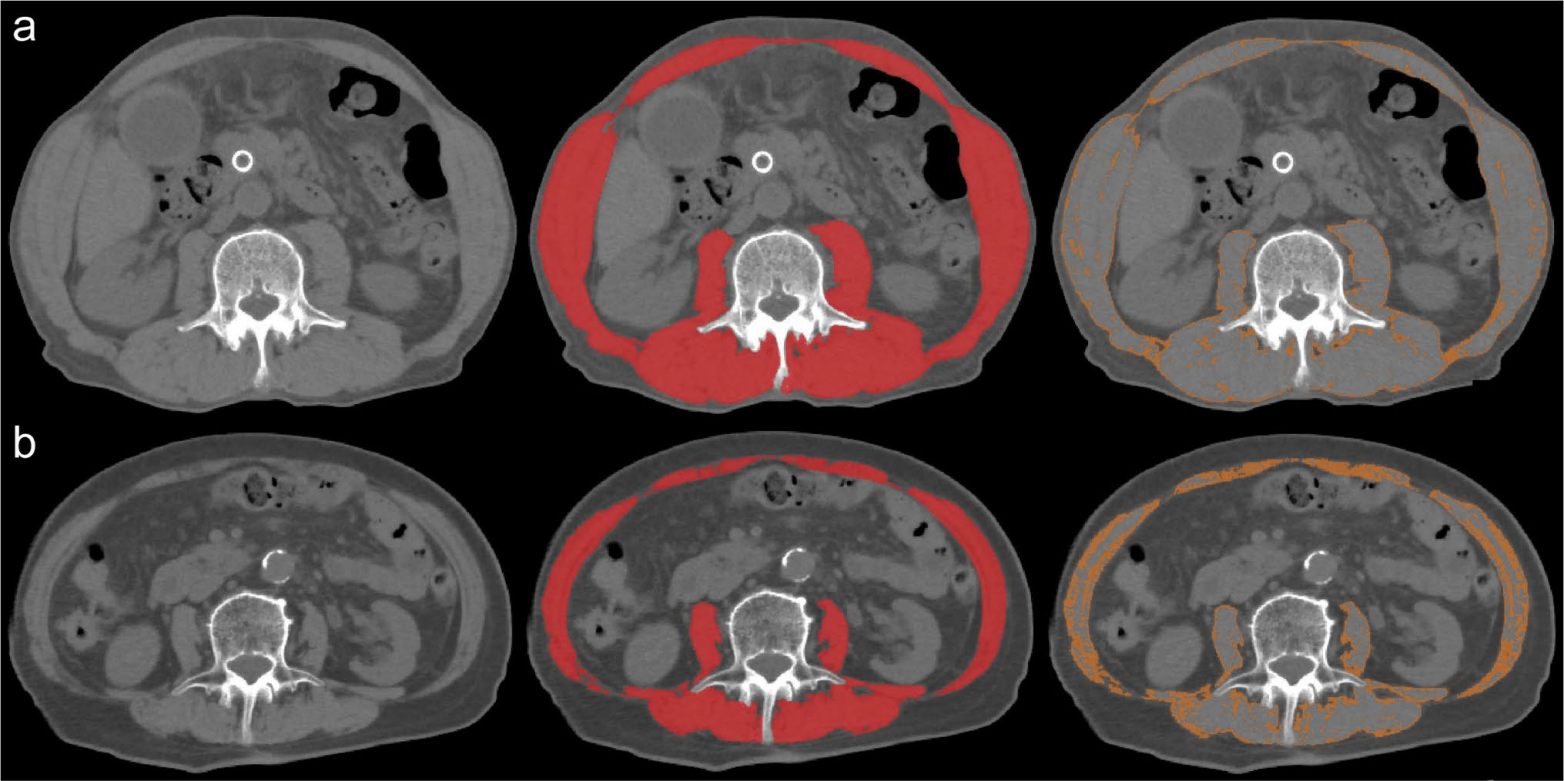
Computed tomography-based assessments of volume and density of skeletal muscles at the level of the third lumbar vertebra [left, non-annotated non-enhanced image; middle, annotated with skeletal muscle area (− 29 to + 150 HU); right, annotated with relatively low-density areas of skeletal muscle (− 29 to + 29 HU) suggesting lipid infiltration]. **a** Case with extrahepatic bile duct cancer showing high levels of skeletal muscle volume and density. **b** Case with extrahepatic bile duct cancer showing low levels of skeletal muscle volume and density. Of note, body mass indexes of the two patients were at the same level (**a**, 20.9 kg/m^2^ and **b**, 21.7 kg/m^2^). *HU* Hounsfield unit

Skeletal muscle volume and density were estimated using sliceOmatic software (version 5.0, TomoVision, Montreal, Canada) on axial-plane CT images at the level of the third lumbar vertebra (Fig. 2) [24]. For each case, a single investigator (STak), blinded to other clinical data, exported an image of a slice with the transverse processes most clearly delineated in the DICOM (Digital Imaging and Communications in Medicine) format and evaluated all skeletal muscle areas included in the image using the software. The muscle areas included the psoas, erector spinae, quadratus lumborum, transversus, abdominis, external and internal obliques, and rectus abdominis. The muscle-fat boundaries were manually outlined. Subsequently, the skeletal muscles were identified based on Hounsfield unit (HU) thresholds of − 29 to + 150. Skeletal muscle index (SMI, cm^2^/m^2^) was calculated as the summed area of the skeletal muscles divided (normalized) by the squared body height. Sarcopenia was defined according to the guidelines of Japan Society of Hepatology [25]: *i.e.*, SMI < 42 cm^2^/m^2^ for males and SMI < 38 cm^2^/m^2^ for females. Skeletal muscle density (SMD) was defined as the mean HU measurement of all skeletal muscles at the same image. Low density of skeletal muscles may reflect increased intramuscular fat contents [26]. Intramuscular adipose tissue content (IMAC) was calculated as the mean HU measurement of all skeletal muscles divided by that of subcutaneous fat (Supplementary Fig. 1) [27]. Subcutaneous fat was identified based on HU thresholds of − 190 to − 30. IMAC has been associated with skeletal muscle functionality [28], and low levels (bottom quartile) of IMAC were examined as a surrogate for decreased functionality in our exploratory analysis.

We evaluated CT images obtained within one month of the diagnosis of bile tract cancer as baseline and additionally evaluated CT images obtained in 2–4 months after the baseline CT for analyses of serial changes of SMI and SMD. Non-enhanced (or contrast-enhanced) CT images were used for the assessment of skeletal muscle volume, and only non-enhanced CT images were used for the assessment of SMD and IMAC considering the increased density of muscles due to the contrast.

### Assessment of chemotherapy outcomes

Among patients receiving chemotherapy, the response to treatment (complete or partial response, stable disease, or progressive disease) was evaluated according to the RECIST [Response Evaluation Criteria in Solid Tumors] version 1.1 [29]. The relative dose intensity (RDI) was defined as a ratio of actual dose administered to the dose specified by the corresponding regimen in the first two months of the chemotherapy initiation. In combination chemotherapy regimens, the overall RDI was calculated as a mean of the RDI of the agents included in the regimen [30, 31]. Adverse events were defined and graded according to the National Cancer Institute Common Terminology Criteria for Adverse Events (version 5.0) [32].

### Statistical analysis

The statistical analyses are detailed in Supplementary Information. We examined associations of skeletal muscle metrics at baseline [sarcopenia (low-level SMI) and SMD] with PFS and overall survival (OS). The Cox proportional hazards regression model was used to calculate hazard ratios (HRs) and 95% confidence intervals (CIs) for PFS and OS according to the skeletal muscle status. The trend was assessed by entering quartile-specific median values of the corresponding muscle metric as a continuous variable in the Cox regression model. To adjust for potential confounding factors, the multivariable Cox regression model initially included the variables described in the corresponding tables. A backward elimination with a threshold *P* value of 0.05 was conducted to select variables for the final model. Cumulative survival probabilities were estimated by the Kaplan–Meier method and compared using the log-rank test for trend. In analyses of a reduction in SMI or SMD as an outcome variable, the multivariable logistic regression model was used.

All statistical analyses were conducted using the Stata software (version 18, StataCorp LLC, College Station, Texas, USA), and all *P* values were two-sided. In our primary analyses, we examined two muscle metrics for two survival outcomes and therefore, used the adjusted α level of 0.013 (*i.e.*, *P* = 0.05/4) for statistical significance based on the Bonferroni correction.

## Results

### Patient characteristics

We included 386 patients with biliary tract cancer, and the cancer status was localized, metastatic, and refractory in 88 (23%), 161 (42%), and 137 (35%) patients, respectively (Fig. 1, Table 1 [33], and Supplementary Table 1). Sarcopenia was associated with female sex and low levels of body mass index (BMI) and performance status. Lower SMD was associated with older age, higher BMI, worse performance status, less advanced cancer status, lower levels of cholinesterase, and higher levels of modified Glasgow prognostic score [34] and neutrophil-to-lymphocyte ratio. There were no differences between sarcopenia and non-sarcopenia patients in terms of treatment outcomes, including treatment response, RDI, and grades 3–4 adverse events. During the median follow-up time of 15.2 months (interquartile range, 10.7–34.8 months) for all censored patients, 348 patients (90% of the total study population) were deceased. Among 291 patients who received chemotherapy, 189 (65%) patients underwent cancer progression.

**Table 1:** Clinical characteristics of patients with advanced biliary tract cancer according to the presence of sarcopenia at baseline or sex-specific quartiles of SMD at baseline

		Sarcopenia^a^ at baseline (n = 386)			SMD at baseline^b^ (n = 359)				
Characteristic^c^	Total (n = 386)	Present (n = 174)	Absent (n = 212)	*P* value	Q1 Lowest (n = 90)	Q2 (n = 90)	Q3 (n = 90)	Q4 Highest (n = 89)	*P* value
Age, years	71.4 ± 11.3	72.4 ± 10.9	70.5 ± 11.6	0.10	77.6 ± 9.7	73.1 ± 9.1	71.8 ± 8.7	63.7 ± 12.7	< 0.001
Sex				< 0.001					0.99
Female	146 (38%)	91 (52%)	55 (26%)		33 (37%)	33 (37%)	33 (37%)	33 (37%)	
Male	240 (62%)	83 (48%)	157 (74%)		57 (63%)	57 (63%)	57 (63%)	56 (63%)	
BMI, kg/m^2^	22.1 ± 3.6	20.3 ± 3.0	23.6 ± 3.3	< 0.001	23.7 ± 3.6	22.5 ± 3.4	21.4 ± 3.1	21.1 ± 3.3	< 0.001
Diabetes mellitus				0.051					0.78
Absent	283 (73%)	136 (78%)	147 (69%)		66 (73%)	65 (72%)	64 (71%)	69 (78%)	
Present	103 (27%)	38 (22%)	65 (31%)		24 (27%)	25 (28%)	26 (29%)	20 (22%)	
ECOG PS				< 0.001					< 0.001
0	161 (42%)	59 (34%)	102 (48%)		29 (32%)	32 (36%)	38 (42%)	47 (53%)	
1	183 (47%)	86 (49%)	97 (46%)		40 (45%)	50 (56%)	44 (49%)	40 (45%)	
2–4	42 (11%)	29 (17%)	13 (6.1%)		21 (23%)	8 (8.9%)	8 (8.9%)	2 (2.2%)	
Appetite loss				0.014					0.060
Absent	277 (72%)	114 (66%)	163 (77%)		54 (60%)	64 (71%)	68 (76%)	68 (76%)	
Present	109 (28%)	60 (34%)	49 (23%)		36 (40%)	26 (29%)	22 (24%)	21 (24%)	
Weight loss^d^				0.13					0.14
Absent	285 (74%)	122 (70%)	163 (77%)		70 (78%)	58 (64%)	70 (78%)	66 (74%)	
Present	101 (26%)	52 (30%)	49 (23%)		20 (22%)	32 (36%)	20 (22%)	23 (26%)	
Primary tumor site				0.20					0.91
Intrahepatic bile duct	105 (27%)	41 (24%)	64 (30%)		25 (28%)	25 (28%)	31 (34%)	21 (24%)	
Extrahepatic bile duct	178 (46%)	85 (49%)	93 (44%)		41 (46%)	42 (47%)	41 (46%)	43 (48%)	
Gallbladder	82 (21%)	35 (20%)	47 (22%)		19 (21%)	19 (21%)	16 (18%)	20 (22%)	
Ampulla	21 (5.4%)	13 (7.5%)	8 (3.8%)		5 (5.6%)	4 (4.4%)	2 (2.2%)	5 (5.6%)	
Cancer status				0.66					0.002
Localized	88 (23%)	43 (25%)	45 (21%)		33 (37%)	22 (24%)	11 (12%)	19 (21%)	
Metastatic	161 (42%)	69 (40%)	92 (43%)		38 (42%)	42 (47%)	44 (49%)	32 (36%)	
Recurrent	137 (35%)	62 (35%)	75 (36%)		19 (21%)	26 (29%)	35 (39%)	38 (43%)	
Treatment				0.34					< 0.001
Gem/CDDP	163 (42%)	72 (41%)	91 (43%)		28 (31%)	36 (40%)	39 (43%)	50 (56%)	
Gem/S-1	27 (7.0%)	10 (5.7%)	17 (8.0%)		2 (2.2%)	10 (11%)	11 (12%)	4 (4.5%)	
Gem	62 (16%)	28 (16%)	34 (16%)		17 (19%)	14 (16%)	19 (21%)	7 (7.9%)	
S-1	24 (6.2%)	11 (6.3%)	13 (6.1%)		3 (3.3%)	2 (2.2%)	7 (7.8%)	5 (5.6%)	
FOLFIRINOX [33]	15 (3.9%)	3 (1.7%)	12 (5.7%)		1 (1.1%)	3 (3.3%)	1 (1.1%)	10 (11%)	
Radiation	13 (3.4%)	7 (4.0%)	6 (2.8%)		3 (3.3%)	2 (2.2%)	3 (3.3%)	5 (5.6%)	
Best supportive care	82 (21%)	43 (25%)	39 (18%)		36 (40%)	23 (26%)	10 (11%)	8 (9.0%)	
CEACAM5 (CEA), ng/mL	4.7 (2.9–11.3)	5.3 (2.9–13.9)	4.6 (2.9–10.2)	0.43	5.3 (3.2–11.4)	4.0 (2.8–9.9)	4.8 (2.7–22.9)	4.5 (2.8–10.3)	0.42
CA19-9, U/mL	183	250	155	0.039	123	260	268	153	0.11
	(32–821)	(47–1028)	(25–679)		(27–780)	(28–1205)	(48–3900)	(33–559)	
mGPS				0.42					< 0.001
0	216 (59%)	91 (55%)	125 (62%)		34 (39%)	45 (52%)	51 (62%)	66 (76%)	
1	31 (8.4%)	15 (9.0%)	16 (7.9%)		4 (4.6%)	9 (10%)	8 (9.8%)	9 (10%)	
2	122 (33%)	60 (36%)	62 (31%)		49 (56%)	33 (38%)	23 (28%)	12 (14%)	
Cholinesterase, U/L	222	209	227	0.028	183	226	217	252	< 0.001
	(167–271)	(153–253)	(180–274)		(129–227)	(180–269)	(170–267)	(192–294)	
Total cholesterol, mg/dL	173	172	176	0.81	156	176	170	190	0.19
	(142–207)	(142–213)	(141–206)		(133–195)	(151–226)	(147–196)	(142–215)	
NLR	3.2 (2.1–4.6)	3.3 (2.1–5.2)	3.2 (2.1–4.3)	0.45	4.2 (2.8–6.3)	3.2 (2.0–5.3)	3.1 (2.0–4.3)	2.6 (2.0–3.8)	< 0.001
Treatment response				0.89					0.60
Partial response	31 (11%)	12 (10%)	19 (12%)		3 (6.1%)	6 (9.7%)	9 (12%)	9 (12%)	
Stable disease	166 (59%)	71 (59%)	95 (58%)		28 (57%)	36 (58%)	43 (57%)	49 (66%)	
Progressive disease	85 (30%)	37 (31%)	48 (30%)		18 (37%)	20 (32%)	23 (31%)	16 (22%)	
Relative dose intensity in the first two months, %	76 (58–89)	76 (52–88)	77 (61–89)	0.51	80 (64–83)	70 (44–85)	79 (62–89)	74 (61–89)	0.39
Grades 3–4 adverse events in the first two months				0.33					0.016
Absent	176 (60%)	71 (57%)	105 (63%)		33 (65%)	29 (45%)	54 (70%)	47 (62%)	
Present	115 (40%)	53 (43%)	62 (37%)		18 (35%)	36 (55%)	23 (30%)	29 (38%)	

^a^Sarcopenia was defined according to the cut-off values for SMI proposed by Japan Society of Hepatology guidelines [25]: SMI < 42 cm^2^/m^2^ for males and SMI < 38 cm^2^/m^2^ for females

^b^The SMD at baseline was categorized into Q1 (18.5–30.4 HU), Q2 (30.5–35.6 HU), Q3 (35.8–39.9 HU), and Q4 (40.0–52.9 HU) for males and Q1 (7.7–22.1 HU), Q2 (22.2–27.6 HU), Q3 (27.7–32.0 HU), and Q4 (32.5–47.5 HU) for females

^c^Data are presented as mean ± standard deviation, median (interquartile range), or number of patients (%). Percentage indicates the proportion of patients with a specific characteristic in all cases or in strata of the presence of sarcopenia or quartiles of SMD at baseline

^d^Weight loss was defined as > 5% loss of body weight within six months prior to the baseline computed tomography evaluation

*BMI* body mass index; *CA19-9* carbohydrate antigen 19–9; *CDDP* cisplatin; *CEA* carcinoembryonic antigen; *ECOG* Eastern Cooperative Oncology Group; *FOLFILINOX* fluorouracil, leucovorin, irinotecan, and oxaliplatin; *Gem* gemcitabine; *HU* Hounsfield unit; *mGPS* modified Glasgow prognostic score; *NLR* neutrophil-to-lymphocyte ratio; *PS* performance status; *Q1-4* quartiles 1–4; *SMD* skeletal muscle density; *SMI* skeletal muscle index

### Skeletal muscle status and survival among patients with advanced biliary tract cancer

**Fig. 3:**
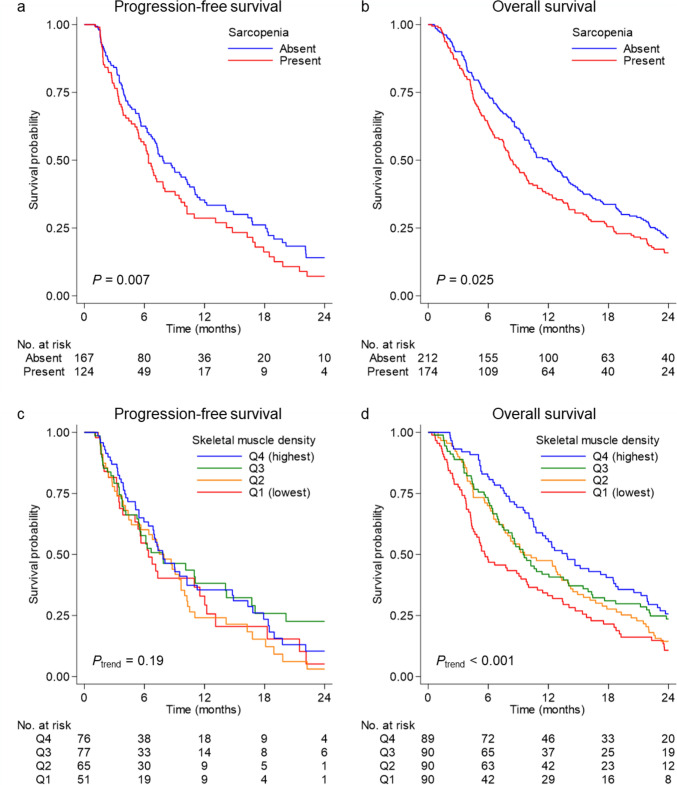
Kaplan–Meier survival curves of patients with advanced biliary tract cancer according to skeletal muscle status. **a** and **b** PFS and OS, respectively, by the presence of sarcopenia at baseline. **c** and **d** PFS and OS, respectively, by SMD at baseline. In patients with and without sarcopenia, the median PFS times were 6.4 (95% CI, 5.4–7.9) and 7.9 months (95% CI, 6.7–10.3 months), respectively, and the median OS times were 8.4 (95% CI, 7.1–9.9) and 11.9 months (95% CI, 10.1–13.9 months), respectively. For the lowest to highest quartiles of SMD, the median PFS times were 6.4 (95% CI, 3.8–11.5), 8.0 (95% CI, 4.4–9.7), 7.9 (95% CI, 5.4–14.1), and 7.8 months (95% CI, 6.4–10.3 months), respectively, and the median OS times were 5.7 (95% CI, 4.4–9.3), 9.6 (95% CI, 7.6–13.3), 9.5 (95% CI, 7.6–13.5), and 13.9 months (95% CI, 10.7–18.5 months), respectively. *CI* confidence interval; *OS* overall survival; *PFS* progression-free survival; *SMD* skeletal muscle density

In our primary analyses (Table 2 and Supplementary Table 2), sarcopenia was associated with short PFS and OS [multivariable HRs, 1.60 (95% CI, 1.15–2.22) and 1.28 (95% CI, 1.03–1.58), respectively] although the association with OS did not reach statistical significance. SMD at baseline was associated with OS (*P*_trend_ = 0.012), but not with PFS (*P*_trend_ = 0.13). The multivariable HR for OS comparing the extreme quartiles of SMD was 1.52 (95% CI, 1.07–2.14). The Kaplan–Meier survival analyses yielded consistent results (Fig. 3). In an exploratory analysis, sarcopenia jointly defined by low levels of SMI and IMAC was associated with short PFS (multivariable HR, 1.80; 95% CI, 1.05–3.08; Supplementary Table 3). Given the differences in SMD values by machines in the current study population (Supplementary Fig. 2), we conducted a survival analysis based on sex-specific quartiles of standard scores (so-called Z scores) of SMD, which yielded similar results to our primary analysis (Supplementary Table 4). In stratified analyses, the association of SMD with OS appeared to be stronger for intrahepatic bile duct or gallbladder cancer than for extrahepatic bile duct cancer (*P*_interaction_ = 0.003, Supplementary Table 5). The survival associations of sarcopenia and SMD did not differ by chemotherapy regimens (*P*_interaction_ > 0.15, Supplementary Table 6). We did not observe a linear association of SMI at baseline with PFS or OS (*P*_trend_ > 0.14, Supplementary Table 7).

**Table 2:** Saropenia and SMD at baseline in relation to survival among patients with advanced biliary tract cancer

	Progression-free survival					Overall survival			
	No. of cases	No. of events	Univariable HR (95% CI)	Multivariable HR^a^ (95% CI)		No. of cases	No. of events	Univariable HR (95% CI)	Multivariable HR^a^ (95% CI)
Sarcopenia^b^ at baseline									
Absent	167	107	1 (referent)	1 (referent)		212	188	1 (referent)	1 (referent)
Present	124	82	1.30 (0.97–1.74)	1.60 (1.15–2.22)		174	160	1.27 (1.03–1.57)	1.28 (1.03–1.58)
*P*			0.075	0.005				0.026	0.027
SMD at baseline									
Q4 (highest)	76	53	1 (referent)	1 (referent)		89	76	1 (referent)	1 (referent)
Q3	77	46	0.96 (0.65–1.43)	1.04 (0.69–1.57)		90	83	1.33 (0.97–1.81)	1.09 (0.78–1.52)
Q2	65	45	1.23 (0.82–1.83)	1.34 (0.89–2.03)		90	84	1.39 (1.02–1.89)	1.04 (0.74–1.45)
Q1 (lowest)	51	32	1.26 (0.81–1.95)	1.46 (0.92–2.33)		90	82	2.03 (1.48–2.79)	1.52 (1.07–2.14)
*P*_trend_^c^			0.24	0.13				0.001	0.012

^a^The multivariable Cox regression model initially included age, sex, body mass index, diabetes mellitus, performance status, primary tumor site, cancer status, receipt of treatment, carbohydrate antigen 19–9, modified Glasgow prognostic score, and neutrophil-to-lymphocyte ratio. A backward elimination with a threshold *P* of 0.05 was conducted to select variables for the final models. The variables that remained in the final models are described in Supplementary Table 2

^b^Sarcopenia was defined according to the cut-off values for SMI proposed by Japan Society of Hepatology guidelines [25]: SMI < 42 cm^2^/m^2^ for males and SMI < 38 cm^2^/m^2^ for females

^c^*P*_trend_ was calculated by entering quartile-specific median values of SMD at baseline (continuous) in the Cox regression model

*CI* confidence interval; *HR* hazard ratio; *Q1-4* quartiles 1–4; *SMD* skeletal muscle density; *SMI* skeletal muscle index

In our secondary analyses of serial changes of skeletal muscle status (Table 3**, **Supplementary Table 8, and Supplementary Fig. 3), SMI change in 2–4 months was associated with OS [multivariable HR comparing the extreme quartiles, 1.66 (95% CI, 1.17–2.36); *P*_trend_ = 0.009], but not with PFS (*P*_trend_ = 0.93). SMD change in 2–4 months was associated with PFS [multivariable HR comparing the extreme quartiles, 1.82 (95% CI, 1.13–2.94); *P*_trend_ = 0.013], but not with OS (*P*_trend_ = 0.57). Patients with higher levels of SMI and SMD at baseline were more likely to undergo decreases in those parameters (Supplementary Fig. 4). Therefore, we conducted survival analyses of serial changes of SMI and SMD stratified by the baseline values, which suggested similar survival associations of decreases in those parameters across the strata (*P*_interaction_ > 0.24, Supplementary Table 9).

**Table 3:** SMI and SMD changes in 2–4 months in relation to survival among patients with advanced biliary tract cancer

	Progression-free survival					Overall survival			
	No. of cases	No. of events	Univariable HR (95% CI)	Multivariable HR^a^ (95% CI)		No. of cases	No. of events	Univariable HR (95% CI)	Multivariable HR^a^ (95% CI)
SMI change in 2–4 months									
Q4 (less loss or gain)	62	43	1 (referent)	1 (referent)		73	67	1 (referent)	1 (referent)
Q3	66	51	1.12 (0.75–1.69)	0.96 (0.63–1.46)		75	67	0.95 (0.68–1.34)	0.85 (0.60–1.21)
Q2	67	53	1.05 (0.70–1.57)	0.97 (0.65–1.45)		74	65	0.86 (0.61–1.21)	0.78 (0.54–1.13)
Q1 (more loss)	60	36	1.08 (0.69–1.69)	0.95 (0.60–1.50)		75	67	1.79 (1.27–2.52)	1.66 (1.17–2.36)
*P*_trend_^b^			0.75	0.93				0.006	0.009
SMD change in 2–4 months									
Q4 (less loss or gain)	55	34	1 (referent)	1 (referent)		61	55	1(referent)	1 (referent)
Q3	54	39	1.21 (0.76–1.91)	1.25 (0.77–2.00)		63	56	1.00 (0.69–1.45)	1.10 (0.74–1.64)
Q2	54	39	1.23 (0.77–1.95)	1.36 (0.84–2.20)		61	52	0.87 (0.59–1.27)	0.86 (0.58–1.29)
Q1 (more loss)	51	41	1.77 (1.12–2.80)	1.82 (1.13–2.94)		63	58	1.35 (0.94–1.96)	1.17 (0.78–1.74)
*P*_trend_^b^			0.015	0.013				0.13	0.57

^a^The multivariable Cox regression model initially included age, sex, body mass index, diabetes mellitus, performance status, primary tumor site, cancer status, receipt of treatment, carbohydrate antigen 19–9, modified Glasgow prognostic score, and neutrophil-to-lymphocyte ratio. A backward elimination with a threshold *P* of 0.05 was conducted to select variables for the final models. The variables that remained in the final models are described in Supplementary Table 8

^b^*P*_trend_ was calculated by entering quartile-specific median values of SMI change or SMD change in 2–4 months (continuous) in the Cox regression model

*CI* confidence interval; *HR* hazard ratio; *Q1-4* quartiles 1–4; *SMD* skeletal muscle density; *SMI* skeletal muscle index

### Prognostic factors for a decrease in skeletal muscle index (SMI)

In secondary analyses, we examined prognostic factors associated with the greatest reduction (the lowest quartile) in SMI or SMD in 2–4 months, which was associated with the worst survival outcomes (Table 4). In a multivariable analysis, progressive disease was associated with the greatest reductions in SMI and SMD with multivariable odds ratios of 2.53 (95% CI, 1.33–4.84) and 2.47 (95% CI, 1.26–4.82), respectively. The greatest reduction in SMI was observed in 37 and 19% patients with progressive disease and disease control, respectively; and that in SMD was observed in 37 and 19% patients with progressive disease and disease control, respectively. There was suggestive evidence for the associations of metastatic disease and cholangitis with the SMI reduction with odds ratios of 1.75 (95% CI, 1.00–3.07) and 1.95 (95% CI, 1.05–3.61), respectively, although these associations did not reach statistical significance at the adjusted α level of 0.013. Given studies suggesting the potential negative impact of sarcopenia on biliary stent patency [35–37], we examined an association of sarcopenia at baseline with the risk of cholangitis in the logistic regression analysis. Sarcopenia at baseline was not associated with cholangitis in 2–4 months (multivariable OR, 1.54; 95% CI, 0.88–2.70; Supplementary Table 10).

**Table 4:** Prognostic factors for the greatest reduction in the SMI or SMD in 2–4 months (quartile 1 vs. quartiles 2–4) among patients with advanced biliary tract cancer (the final multivariable models)

	SMI reduction (n = 297)					SMD reduction (n = 248)			
	No. of cases	No. of events	Multivariable OR^a^ (95% CI)	*P* value		No. of cases	No. of events	Multivariable OR^a^ (95% CI)	*P* value
Cancer status									
Localized	178	39	1 (referent)					Did not remain	
Metastatic	119	36	1.75 (1.00–3.07)	0.050				in this model	
Biliary drainage									
No drainage	161	36	1 (referent)					Did not remain	
Drainage without cholangitis	60	11	0.75 (0.35–1.65)	0.48				in this model	
Drainage with cholangitis	76	28	1.95 (1.05–3.61)	0.034					
Treatment response^b^									
Partial response / stable disease	190	36	1 (referent)			157	30	1 (referent)	
Progressive disease	65	24	2.53 (1.33–4.84)	0.005		57	21	2.47 (1.26–4.82)	0.008

^a^The multivariable logistic regression model initially included age, sex, body mass index, diabetes mellitus, performance status, primary tumor site, cancer status, receipt of treatment, carbohydrate antigen 19–9, modified Glasgow prognostic score, neutrophil-to-lymphocyte ratio, biliary drainage status, and treatment response. A backward elimination with a threshold *P* of 0.05 was conducted to select variables for the final model

^b^To calculate ORs for treatment response, we excluded cases receiving radiation or best supportive care. To calculate ORs for other factors, we assigned stable disease for cases receiving radiation (all these cases did not undergo cancer progression in 2–4 months), and progressive disease for cases receiving best supportive care

*CI* confidence interval; *OR* odds ratio; *SMD* skeletal muscle density; *SMI* skeletal muscle index

## Discussion

In this retrospective cohort study, we have shown that sarcopenia and SMD at baseline may be associated with survival outcomes of patients with advanced biliary tract cancer. In addition, our data support survival associations of serial reductions in those parameters. Interestingly, our survival analysis of serial muscular changes stratified by the baseline values suggests the prognostic relevance of decreases in SMI and SMD irrespective of the baseline status. Cholangitis as well as aggressive tumor characteristics appeared to predispose the patients to high risk of unfavorable muscular alterations. Our findings support an important role of skeletal muscle quality and quantity in determining survival outcomes of patients with biliary tract cancer.

In the current study, sarcopenia and low-level skeletal muscle density were associated with worse survival outcomes among patients with advanced biliary tract cancer. Given no differences in treatment responses and RDI between the subgroups defined by baseline muscular status, it was considered that the duration of treatment response may be shortened by unfavorable skeletal muscular status and resultant systemic changes (*e.g.*, inflammation and impaired immune status) rather than decreased tolerability to chemotherapy. Evidence suggests that skeletal muscle status may represent a mutually dependent relationship with systemic inflammatory and immune status [38]. Sarcopenia presenting as skeletal muscle depletion not only occurs as a consequence of cancer-related inflammation and malnutrition, but also impair anti-cancer immunity [39]. Under the presence of skeletal muscle depletion, adaptive and innate immune responses are attenuated via dysregulations of various immune parameters such as the dysfunction of natural killer cells via myokines and adipokines [40], potentially resulting in tumor progression and poor clinical outcomes. We also found an inverse association of SMD and mortality hazards. Decreased levels of muscle density estimated by muscle radiation attenuation may reflect increased infiltration of lipid contents into intra- and inter-muscular regions [26]. Myosteatosis potentially provokes hyperinsulinemia and insulin resistance independently of the total body fatness [41]. Mechanistic studies have shown that hyperinsulinemia increases the bioavailability of IGF1 (insulin-like growth factor 1) and up-regulates cancer-related signaling pathways such as the PI3K-AKT-MTOR pathway [42]. Insulin resistance may be correlated with systemic inflammation [43] and thereby, promote tumor growth. The null findings on the prognostic roles of skeletal muscular status in some survival outcomes require comments. Given the trends in stratum-specific hazard ratios in the analysis of the baseline values (Table 2), there might be a possibility of type II errors due to the limited sample size (false negative findings). On the other hand, there were no trends in the associations of serial changes of SMI and SMD with PFS and OS, respectively (Table 3). We speculate that SMD alterations may play an important role in the duration of the first-line chemotherapy whereas SMI alterations may have a greater impact on the duration of later-line chemotherapy regimens. However, a larger study is warranted to validate these speculations. Physical exercise may improve skeletal muscle status [44, 45], prevent surgical complications [46], and prolong survival times [47] in cancer patients undergoing radical treatment. Given our data suggesting the prognostic impact of skeletal muscle deterioration in patients with advanced biliary tract cancer, clinical research is desired to examine whether this patient population can obtain survival benefits from physical exercise programs and pharmacological treatment for skeletal muscles [48, 49].

Risk factors for a temporal reduction in skeletal muscle quantity or quality have not been investigated in patients with biliary tract cancer. In the current study, advanced cancer status (progressive or metastatic disease) and cholangitis were independent risk factors for premature deterioration of skeletal muscle status, which was associated with worse outcomes in our survival analyses. Given the evidence linking cancer development and progression to sarcopenia, the associations of advanced cancer status with skeletal muscle loss were considered plausible. Patients with biliary tract cancer may repeatedly develop bacterial cholangitis due to biliary obstruction or reflux of the intestinal contents [50]. Clinical studies have shown that patients with obstructive jaundice are predisposed to systemic inflammatory reactions characterized by high levels of pro-inflammatory cytokines [*e.g.*, IL6 and TNF (tumor necrosis factor, TNF-α)] [51]. Elevated levels of these cytokines may be observed after successful biliary drainage at least in part due to subclinical cholangitis [52], suggesting sustained systemic inflammation in patients with biliary obstruction. Further evidence suggests that cholestasis may promote bacterial translocation due to disrupted integrity of the gastrointestinal mucosa [53], potentially contributing to chronic inflammation and muscle wasting. Research is warranted to explore the mechanism through which cholangitis results in skeletal muscle depletion and to establish biliary drainage strategies that can minimize the risk of cholangitis for prolonged survival of patients with advanced biliary tract cancer.

We acknowledge limitations of the current study. First, there might be unmeasured confounding factors in our survival analyses. However, the adjustment for a variety of clinical characteristics did not alter our findings materially. Second, a lack of gold-standard measurement methods for skeletal muscle status is another limitation; nonetheless, it is more likely that misclassifications of those parameter would have driven the survival associations of skeletal muscle status towards the null hypothesis. Third, our definition of sarcopenia was solely based on the assessment of skeletal muscle volume. Given the importance of muscle strength in assessing the sarcopenic status [54–56], our survival data should be validated using a dataset integrated with the metrics of skeletal muscle functionality. Fourth, despite the null association of sarcopenia at baseline with the risk of cholangitis in our study, there might be a possibility of a reverse causation in our conclusion on the association of cholangitis with muscular status. Functional studies are required to make a definite conclusion on the causality.

In conclusion, our data suggest that skeletal muscle status and its serial alteration may have prognostic relevance in patients with advanced biliary tract cancer. Further research is warranted to examine whether interventions aimed at improvements of skeletal muscle quality and quantity can prolong survival of patients with this lethal malignancy. In addition to a metastatic or progressive disease, cholangitis may exert a negative impact on skeletal muscle volume, and therefore, biliary stenting strategies should be optimized to minimize the risk of cholangitis in management of patients with biliary tract cancer.

### Supplementary Information

Below is the link to the electronic supplementary material.

Supplementary file1 (DOCX 1678 KB)

## Abbreviations

BMI: Body mass index
CI: Confidence interval
CT: Computed tomography
HR: Hazard ratio
HU: Hounsfield unit
IMAC: Intramuscular adipose tissue content
OS: Overall survival
PFS: Progression-free survival
RDI: Relative dose intensity
SMD: Skeletal muscle density
SMI: Skeletal muscle index

## Declarations

### Data availability

Data are available from the corresponding author on reasonable request.
