# Supplementary material for: Skeletal muscle status and survival among patients with advanced biliary tract cancer
Source: Int J Clin Oncol. 2024 Feb 6;29(3):297–308. doi: 10.1007/s10147-023-02466-z (PMC10884055; doi:10.1007/s10147-023-02466-z)
Supplement: Supplementary file 1 — Supplementary file1 (DOCX 1678 KB) [file 10147_2023_2466_MOESM1_ESM.docx]

**Supplementary Information**

**Article title**

Skeletal muscle status and survival among patients with advanced biliary tract cancer

**Journal name**

International Journal of Clinical Oncology

**Author names and affiliations**

Shinya Takaoka^1^, Tsuyoshi Hamada^1,2^, Naminatsu Takahara^1^, Kei Saito^1^, Go Endo^1^, Ryunosuke Hakuta^1^,

Kota Ishida^1^, Kazunaga Ishigaki^1^, Sachiko Kanai^1,3^, Kohei Kurihara^1^, Hiroki Oyama^1^, Tomotaka Saito^1^,

Tatsuya Sato^1^, Tatsunori Suzuki^1^, Yukari Suzuki^1^, Shuichi Tange^1^, Yurie Tokito^1^, Ryosuke Tateishi^1^,

Yousuke Nakai^1,3^, Mitsuhiro Fujishiro^1^

^1^ Department of Gastroenterology, Graduate School of Medicine, The University of Tokyo, Tokyo, Japan

^2^ Department of Hepato-Biliary-Pancreatic Medicine, The Cancer Institute Hospital, Japanese Foundation for Cancer Research, Tokyo, Japan

^3^ Department of Endoscopy and Endoscopic Surgery, The University of Tokyo Hospital, Tokyo, Japan

**Corresponding author**

Yousuke Nakai, MD, PhD

Department of Endoscopy and Endoscopic Surgery,

The University of Tokyo Hospital

7-3-1 Hongo, Bunkyo City, Tokyo 113-8655, Japan

Tel: +81-3-3815-5411

Fax: +81-3-3814-0021

E-mail: ynakai-tky@umin.ac.jp

**Abbreviations:** CA19-9, carbohydrate antigen 19-9; CI, confidence interval; CRP, C-reactive protein; HR, hazard ratio; mGPS, modified Glasgow prognostic score; NLR, neutrophil-to-lymphocyte ratio; OS, overall survival; PFS, progression-free survival; SMD, skeletal muscle density; SMI, skeletal muscle index.

**Statistical analysis**

In our primary analyses, we examined associations of sarcopenia (low-level skeletal muscle index [SMI]) and skeletal muscle density (SMD) with progression-free survival (PFS) and overall survival (OS) among patients with biliary tract cancer. The investigations of serial changes of skeletal muscle metrics and stratum-specific risk estimates represented secondary analyses. PFS was defined as time from the diagnosis to the first documentation of progressive disease or death of any cause, whichever came first. When any of these endpoints was not observed, the patients were censored at the time-point of the last cross-sectional imaging study. OS was defined as time from the diagnosis to death of any cause, where patients who were alive at the last follow-up were censored. The Cox proportional hazards regression model was used to calculate hazard ratios (HRs) and 95% confidence interval (CIs) for PFS and OS according to the skeletal muscle status. The trend was assessed by entering quartile-specific median values of the corresponding muscle metric as a continuous variable in the Cox regression model and evaluating the Wald test. To adjust for potential confounding factors, the multivariable Cox regression model initially included the following variables: age at diagnosis (continuous), sex (female vs. male), body mass index (< 18.5 vs. 18.5-25.0 vs. > 25.0 kg/m^2^), diabetes mellitus (absent vs. present), performance status (0 vs. 1-4), primary tumor site (intrahepatic bile duct vs. extrahepatic bile duct vs. gallbladder vs. ampulla), cancer status (localized vs. metastatic), receipt of treatment (combination chemotherapy vs. single agent chemotherapy vs. radiation vs. best supportive care), carbohydrate antigen 19-9 (CA19-9, quartiles, categorical), modified Glasgow prognostic score (mGPS, 0 vs. 1 vs. 2), and neutrophil-to-lymphocyte ratio (NLR, quartiles, categorical). The mGPS has been correlated with systemic inflammatory response, nutritional status, and patient survival in a variety of cancer types [1], and was defined as 0 for patients with C-reactive protein (CRP) ≤ 1.0 mg/dL, 1 for patients with CRP > 1.0 mg/dL and albumin ≥ 3.5 g/dL, and 2 for patients with CRP > 1.0 mg/dL and albumin < 3.5 g/dL [2]. For cases with missing data on CA19-9 (2.3%), mGPS (4.4%), and NLR (4.9%), we assigned median values for CA19-9 and NLR and the major category for mGPS. We confirmed that the exclusion of cases with missing data did not change our findings substantially (data not shown). A backward elimination with a threshold *P* value of 0.05 was conducted to select variables for the final model. Taking the multicollinearity into account, we did not include carcinoembryonic antigen, cholinesterase, and total cholesterol in the multivariable models. In stratified analyses, a statistical interaction was assessed by entering main effect terms and a cross-product term of sarcopenia or SMD (quartile-specific medians) and a stratification variable (tumor site or chemotherapy regimen) into the model and evaluating the likelihood ratio test. We calculated stratum-specific HRs by sarcopenia or SMD based on a single regression model with a re-parameterization of the interaction term [3]. The Kaplan-Meier method was used to estimate cumulative survival probabilities with stratum-specific median times. The cumulative survival probabilities were compared using the log-rank test for trend. In analyses of a reduction in SMI or SMD as an outcome variable, the multivariable logistic regression model included treatment response (partial response or stable disease vs. progressive disease) and biliary drainage status in 2-4 months (no drainage vs. drainage without cholangitis vs. drainage with cholangitis) in addition to the same set of the covariates in the Cox regression model. Cholangitis in 2-4 months of chemotherapy initiation (after the baseline assessments of SMI and SMD) was defined when biliary drainage was performed for cholangitis defined by the Tokyo Guidelines 2018 [4]. To compare characteristics between subgroups, we used the chi-square test or the Fisher’s exact test, as appropriate, for categorical variables, and the analysis of variance or the Kruskal-Wallis test, as appropriate, for continuous variables.

All statistical analyses were conducted using the Stata software (version 18, StataCorp LLC, College Station, Texas, USA), and all *P* values were two-sided. In our primary analyses, we examined two muscle metrics for two survival outcomes and therefore, used the adjusted α level of 0.013 (*i.e.*, *P* = 0.05/4) for statistical significance based on the Bonferroni correction. The results of secondary analyses were interpreted cautiously in addition to the use of the adjusted α level of 0.013.

**Supplementary Table 1** Clinical characteristics of patients with advanced biliary tract cancer according to sex-specific quartiles of SMI change or SMD change in 2-4 months

|  |  | SMI change in 2-4 months^a^ (n=297) | | | |  | SMD change in 2-4 months^a^ (n=248) | | | |  |
| --- | --- | --- | --- | --- | --- | --- | --- | --- | --- | --- | --- |
| Characteristic^b^ | Total  (n=297) | Q1  More loss  (n=75) | Q2  (n=74) | Q3  (n=75) | Q4  Less loss or gain  (n=73) | *P* value | Q1  More loss  (n=63) | Q2  (n=61) | Q3  (n=63) | Q4  Less loss or gain  (n=61) | *P* value |
| Age, years | 70.2 ± 10.8 | 71.8 ± 9.8 | 70.0 ± 9.4 | 69.1 ± 10.7 | 70.1 ± 12.9 | 0.48 | 72.0 ± 9.7 | 67.2 ± 12.0 | 69.7 ± 12.7 | 72.1 ± 9.0 | 0.048 |
|  |  |  |  |  |  |  |  |  |  |  |  |
| Sex |  |  |  |  |  | 0.99 |  |  |  |  | 0.99 |
| Female | 114 (38%) | 29 (39%) | 28 (38%) | 29 (39%) | 28 (38%) |  | 24 (38%) | 23 (38%) | 24 (38%) | 23 (38%) |  |
| Male | 183 (62%) | 46 (61%) | 46 (62%) | 46 (61%) | 45 (62%) |  | 39 (62%) | 38 (62%) | 39 (62%) | 38 (62%) |  |
|  |  |  |  |  |  |  |  |  |  |  |  |
| BMI, kg/m^2^ | 22.1 ± 3.5 | 22.1 ± 3.9 | 22.4 ± 2.9 | 22.7 ± 3.9 | 21.5 ± 3.1 | 0.20 | 22.4 ± 3.3 | 21.8 ± 3.2 | 22.3 ± 3.8 | 21.9 ± 3.4 | 0.72 |
|  |  |  |  |  |  |  |  |  |  |  |  |
| Diabetes mellitus |  |  |  |  |  | 0.64 |  |  |  |  | 0.23 |
| Absent | 221 (74%) | 58 (77%) | 53 (72%) | 53 (71%) | 57 (78%) |  | 42 (67%) | 50 (82%) | 49 (78%) | 47 (77%) |  |
| Present | 76 (26%) | 17 (23%) | 21 (28%) | 22 (29%) | 16 (22%) |  | 21 (33%) | 11 (18%) | 14 (22%) | 14 (23%) |  |
|  |  |  |  |  |  |  |  |  |  |  |  |
| ECOG PS |  |  |  |  |  | 0.13 |  |  |  |  | 0.74 |
| 0 | 133 (45%) | 31 (41%) | 34 (46%) | 42 (56%) | 26 (36%) |  | 25 (40%) | 32 (52%) | 30 (48%) | 24 (39%) |  |
| 1 | 146 (49%) | 39 (52%) | 37 (50%) | 31 (41%) | 39 (53%) |  | 33 (52%) | 26 (43%) | 30 (48%) | 32 (52%) |  |
| 2-4 | 18 (6.1%) | 5 (6.7%) | 3 (4.1%) | 2 (2.7%) | 8 (11%) |  | 5 (7.9%) | 3 (4.9%) | 3 (4.8%) | 5 (8.2%) |  |
|  |  |  |  |  |  |  |  |  |  |  |  |
| Appetite loss |  |  |  |  |  | 0.090 |  |  |  |  | 0.15 |
| Absent | 222 (75%) | 48 (64%) | 59 (80%) | 57 (76%) | 58 (79%) |  | 45 (71%) | 51 (84%) | 47 (75%) | 40 (66%) |  |
| Present | 75 (25%) | 27 (36%) | 15 (20%) | 18 (24%) | 15 (21%) |  | 18 (29%) | 10 (16%) | 16 (25%) | 21 (34%) |  |
|  |  |  |  |  |  |  |  |  |  |  |  |
| Weight loss^c^ |  |  |  |  |  | 0.079 |  |  |  |  | 0.91 |
| Absent | 224 (75%) | 52 (69%) | 60 (81%) | 62 (83%) | 50 (68%) |  | 48 (76%) | 47 (77%) | 46 (73%) | 44 (72%) |  |
| Present | 73 (25%) | 23 (31%) | 14 (19%) | 13 (17%) | 23 (32%) |  | 15 (24%) | 14 (23%) | 17 (27%) | 17 (28%) |  |
|  |  |  |  |  |  |  |  |  |  |  |  |
| Primary tumor site |  |  |  |  |  | 0.99 |  |  |  |  | 0.62 |
| Intrahepatic bile duct | 82 (28%) | 21 (28%) | 21 (28%) | 22 (29%) | 18 (25%) |  | 21 (33%) | 16 (26%) | 16 (25%) | 21 (34%) |  |
| Extrahepatic bile duct | 143 (48%) | 36 (48%) | 33 (45%) | 36 (48%) | 38 (52%) |  | 25 (40%) | 28 (46%) | 33 (52%) | 31 (51%) |  |
| Gallbladder | 60 (20%) | 15 (20%) | 16 (22%) | 14 (19%) | 15 (21%) |  | 16 (25%) | 14 (23%) | 12 (19%) | 7 (11%) |  |
| Ampulla | 12 (4.0%) | 3 (4.0%) | 4 (5.4%) | 3 (4.0%) | 2 (2.7%) |  | 1 (1.6%) | 3 (4.9%) | 2 (3.2%) | 2 (3.3%) |  |
|  |  |  |  |  |  |  |  |  |  |  |  |
|  |  |  |  |  |  |  |  |  |  |  |  |
| Cancer status |  |  |  |  |  | 0.071 |  |  |  |  | 0.42 |
| Localized | 62 (21%) | 17 (23%) | 13 (18%) | 13 (17%) | 19 (26%) |  | 12 (19%) | 14 (23%) | 12 (19%) | 16 (26%) |  |
| Metastatic | 119 (40%) | 36 (48%) | 33 (45%) | 22 (29%) | 28 (38%) |  | 30 (48%) | 19 (31%) | 29 (46%) | 27 (44%) |  |
| Recurrent | 116 (39%) | 22 (29%) | 28 (38%) | 40 (53%) | 26 (36%) |  | 21 (33%) | 28 (46%) | 22 (35%) | 18 (30%) |  |
|  |  |  |  |  |  |  |  |  |  |  |  |
| Treatment |  |  |  |  |  | 0.037 |  |  |  |  | 0.34 |
| Gem/CDDP | 144 (48%) | 33 (44%) | 39 (53%) | 36 (48%) | 35 (48%) |  | 28 (44%) | 32 (52%) | 28 (44%) | 31 (51%) |  |
| Gem/S-1 | 21 (7.1%) | 5 (6.7%) | 9 (12%) | 2 (2.7%) | 5 (6.8%) |  | 3 (4.8%) | 4 (6.6%) | 7 (11%) | 3 (4.9%) |  |
| Gem | 55 (19%) | 15 (20%) | 9 (12%) | 15 (20%) | 17 (23%) |  | 15 (24%) | 8 (13%) | 12 (19%) | 15 (25%) |  |
| S-1 | 20 (6.7%) | 4 (5.3%) | 3 (4.1%) | 8 (11%) | 5 (6.8%) |  | 2 (3.2%) | 3 (4.9%) | 4 (6.3%) | 4 (6.6%) |  |
| FOLFIRINOX [5] | 15 (5.1%) | 3 (4.0%) | 7 (9.5%) | 5 (6.7%) | 0 (0%) |  | 3 (4.8%) | 7 (11%) | 3 (4.8%) | 2 (3.3%) |  |
| Radiation | 12 (4.0%) | 1 (1.3%) | 2 (2.7%) | 3 (4.0%) | 6 (8.2%) |  | 1 (1.6%) | 2 (3.3%) | 5 (7.9%) | 1 (1.6%) |  |
| Best supportive care | 30 (10%) | 14 (19%) | 5 (6.8%) | 6 (8.0%) | 5 (6.8%) |  | 11 (17%) | 5 (8.2%) | 4 (6.3%) | 5 (8.2%) |  |
|  |  |  |  |  |  |  |  |  |  |  |  |
| CEACAM5 (CEA), ng/mL | 4.6 (2.9-10.9) | 5.5 (3.5-17.1) | 5.3 (3.0-11.6) | 4.0 (2.8-8.1) | 4.3 (2.8-7.8) | 0.15 | 7.1 (3.3-33.4) | 5.6 (2.8-11.9) | 3.9 (2.9-6.6) | 4.7 (3.0-10.9) | 0.048 |
|  |  |  |  |  |  |  |  |  |  |  |  |
| CA19-9, U/mL | 179 | 276 | 93 | 89 | 264 | 0.14 | 259 | 221 | 73 | 371 | 0.11 |
|  | (29-775) | (50-1815) | (25-788) | (30-619) | (48-722) |  | (25-1348) | (24-743) | (27-630) | (57-1318) |  |
|  |  |  |  |  |  |  |  |  |  |  |  |
| mGPS |  |  |  |  |  | 0.006 |  |  |  |  | 0.21 |
| 0 | 186 (65%) | 36 (50%) | 45 (63%) | 53 (73%) | 52 (73%) |  | 37 (60%) | 40 (70%) | 41 (66%) | 33 (57%) |  |
| 1 | 26 (9.1%) | 8 (11%) | 6 (8.5%) | 10 (14%) | 2 (2.8%) |  | 10 (16%) | 5 (8.8%) | 2 (3.2%) | 6 (10%) |  |
| 2 | 75 (26%) | 28 (39%) | 20 (28%) | 10 (14%) | 17 (24%) |  | 15 (24%) | 12 (21%) | 19 (31%) | 19 (33%) |  |
|  |  |  |  |  |  |  |  |  |  |  |  |
| Cholinesterase, U/L | 228 | 226 | 229 | 259 | 193 | <0.001 | 229 | 246 | 223 | 206 | 0.49 |
|  | (185-277) | (177-279) | (193-271) | (226-314) | (147-243) |  | (192-279) | (181-279) | (185-284) | (179-244) |  |
|  |  |  |  |  |  |  |  |  |  |  |  |
| Total cholesterol, mg/dL | 176 | 168 | 169 | 187 | 195 | 0.48 | 191 | 159 | 164 | 185 | 0.40 |
|  | (144-211) | (144-198) | (132-207) | (155-216) | (147-223) |  | (152-218) | (142-206) | (134-203) | (147-215) |  |
|  |  |  |  |  |  |  |  |  |  |  |  |
| NLR | 2.9 (2.0-4.3) | 3.3 (2.3-4.3) | 2.6 (2.0-4.4) | 3.0 (2.1-4.2) | 2.5 (1.8-4.3) | 0.57 | 3.3 (2.1-4.3) | 3.1 (2.3-4.2) | 2.6 (2.1-3.8) | 2.8 (1.8-4.3) | 0.61 |
|  |  |  |  |  |  |  |  |  |  |  |  |
| Biliary drainage in 2-4 months |  |  |  |  |  | 0.072 |  |  |  |  | 0.047 |
| Absent | 161 (54%) | 36 (48%) | 45 (61%) | 47 (63%) | 33 (45%) |  | 31 (49%) | 41 (67%) | 33 (52%) | 26 (43%) |  |
| Present | 136 (46%) | 39 (52%) | 29 (39%) | 28 (37%) | 40 (55%) |  | 32 (51%) | 20 (33%) | 30 (48%) | 35 (57%) |  |
|  |  |  |  |  |  |  |  |  |  |  |  |
| Cholangitis in 2-4 months |  |  |  |  |  | 0.027 |  |  |  |  | 0.30 |
| Absent | 221 (74%) | 47 (63%) | 62 (84%) | 58 (77%) | 54 (74%) |  | 41 (65%) | 47 (77%) | 49 (78%) | 47 (77%) |  |
| Present | 76 (26%) | 28 (37%) | 12 (16%) | 17 (23%) | 19 (26%) |  | 22 (35%) | 14 (23%) | 14 (22%) | 14 (23%) |  |
|  |  |  |  |  |  |  |  |  |  |  |  |
| Treatment response |  |  |  |  |  | 0.073 |  |  |  |  | 0.064 |
| Partial response | 29 (11%) | 7 (12%) | 9 (13%) | 9 (14%) | 4 (6.5%) |  | 2 (3.9%) | 7 (13%) | 9 (17%) | 5 (9.1%) |  |
| Stable disease | 161 (63%) | 29 (48%) | 43 (64%) | 44 (67%) | 45 (73%) |  | 28 (55%) | 32 (59%) | 35 (64%) | 39 (71%) |  |
| Progressive disease | 65 (25%) | 24 (40%) | 15 (22%) | 13 (20%) | 13 (21%) |  | 21 (41%) | 15 (28%) | 10 (19%) | 11 (20%) |  |
|  |  |  |  |  |  |  |  |  |  |  |  |
| Relative dose intensity in the first two months, % | 77 (62-89) | 74 (61-85) | 76 (62-89) | 79 (59-93) | 79 (66-91) | 0.41 | 78 (57-88) | 77 (61-90) | 76 (64-88) | 81 (70-91) | 0.78 |
|  |  |  |  |  |  |  |  |  |  |  |  |
| Grades 3-4 adverse events in the first two months |  |  |  |  |  | 0.071 |  |  |  |  | 0.36 |
| Absent | 156 (61%) | 38 (63%) | 49 (73%) | 36 (55%) | 33 (53%) |  | 33 (65%) | 38 (70%) | 32 (59%) | 30 (55%) |  |
| Present | 99 (39%) | 22 (37%) | 18 (27%) | 30 (45%) | 29 (47%) |  | 18 (35%) | 16 (30%) | 22 (41%) | 25 (45%) |  |
|  |  |  |  |  |  |  |  |  |  |  |  |
| Time between baseline  and follow-up CT, months | 2.5 (2.0-3.3) | 2.6 (2.0-3.3) | 2.3 (2.0-2.8) | 2.8 (2.1-3.4) | 2.7 (2.1-3.5) | 0.023 | 2.4 (2.0-3.0) | 2.4 (2.1-3.0) | 2.5 (2.1-3.5) | 2.7 (2.1-3.4) | 0.39 |
|  |  |  |  |  |  |  |  |  |  |  |  |

^a^ SMI change in 2-4 months was categorized into Q1 (-33.0 to -11.1%), Q2 (-11.0 to -5.6%), Q3 (-5.5 to -0.2%), and Q4 (-0.1 to +16.0%) for males and Q1 (-18.7 to -7.7%), Q2 (-7.6 to -3.1%), Q3 (-3.0 to +2.4%), and Q4 (+2.5 to +34.0%) for females. SMD change in 2-4 months was categorized into Q1 (-31.1 to -8.8%), Q2 (-8.1 to -1.6%), Q3 (-1.2 to +4.2%), and Q4 (+4.3 to +31.6%) for males and Q1 (-60.5 to -8.4%), Q2 (-8.1 to -0.8%), Q3 (-0.4 to +6.2%), and Q4 (+6.7 to +66.3%) for females.

^b^ Data are presented as mean ± standard deviation, median (interquartile range), or number of patients (%). Percentage indicates the proportion of patients with a specific characteristic in all cases or in strata of quartiles of SMI change or SMD change in 2-4 months.

^c^ Weight loss was defined as > 5% loss of body weight within six months prior to the baseline CT evaluation.

Abbreviations: BMI, body mass index; CA19-9, carbohydrate antigen 19-9; CDDP, cisplatin; CEA, carcinoembryonic antigen; CT, computed tomography; ECOG, Eastern Cooperative Oncology Group; FOLFILINOX, fluorouracil, leucovorin, irinotecan, and oxaliplatin; Gem, gemcitabine; mGPS, modified Glasgow prognostic score; NLR, neutrophil-to-lymphocyte ratio; PS, performance status; Q1-4, quartiles 1-4; SMD, skeletal muscle density; SMI, skeletal muscle index

**Supplementary Table 2**  Sarcopenia and SMD at baseline in relation to survival among patients with advanced biliary tract cancer (the final multivariable models)

|  | Multivariable HR^a^ (95% CI) | |  |  | Multivariable HR^a^ (95% CI) | |
| --- | --- | --- | --- | --- | --- | --- |
|  | Progression-free survival | Overall survival |  |  | Progression-free survival | Overall survival |
| Sarcopenia^b^ at baseline |  |  |  | SMD at baseline |  |  |
| Absent | 1 (referent) | 1 (referent) |  | Q4 (highest) | 1 (referent) | 1 (referent) |
| Present | 1.60 (1.15-2.22) | 1.28 (1.03-1.58) |  | Q3 | 1.04 (0.69-1.57) | 1.09 (0.78-1.52) |
|  |  |  |  | Q2 | 1.34 (0.89-2.03) | 1.04 (0.74-1.45) |
|  |  |  |  | Q1 (lowest) | 1.46 (0.92-2.33) | 1.52 (1.07-2.14) |
|  |  |  |  |  |  |  |
| ECOG PS |  |  |  |  |  |  |
| 0 | Did not remain | 1 (referent) |  |  | Did not remain | 1 (referent) |
| 1-4 | in this model | 1.49 (1.19-1.87) |  |  | in this model | 1.49 (1.18-1.89) |
|  |  |  |  |  |  |  |
| Primary tumor site |  |  |  |  |  |  |
| Intrahepatic | 1 (referent) | 1 (referent) |  |  | 1 (referent) | 1 (referent) |
| Extrahepatic | 1.02 (0.71-1.47) | 0.96 (0.71-1.29) |  |  | 0.85 (0.59-1.23) | 0.95 (0.70-1.28) |
| Gallbladder | 2.22 (1.45-3.38) | 1.63 (1.19-2.24) |  |  | 1.88 (1.23-2.88) | 1.54 (1.11-2.15) |
| Ampulla | 2.24 (1.10-4.58) | 0.69 (0.40-1.19) |  |  | 1.28 (0.54-3.04) | 0.61 (0.33-1.13) |
|  |  |  |  |  |  |  |
| Cancer status |  |  |  |  |  |  |
| Localized | Did not remain | 1 (referent) |  |  | Did not remain | 1 (referent) |
| Metastatic | in this model | 1.44 (1.11-1.87) |  |  | in this model | 1.54 (1.18-2.02) |
|  |  |  |  |  |  |  |
| Treatment |  |  |  |  |  |  |
| Combination chemotherapy | Did not remain | 1 (referent) |  |  | Did not remain | 1 (referent) |
| Single agent chemotherapy | in this model | 1.24 (0.94-1.64) |  |  | in this model | 1.15 (0.85-1.56) |
| Radiation |  | 0.55 (0.27-1.12) |  |  |  | 0.57 (0.28-1.15) |
| Best supportive care |  | 2.53 (1.84-3.48) |  |  |  | 2.51 (1.79-3.53) |
|  |  |  |  |  |  |  |
| CA19-9 |  |  |  |  |  |  |
| Q1 (lowest) | Did not remain | 1 (referent) |  |  | Did not remain | 1 (referent) |
| Q2 | in this model | 0.90 (0.66-1.22) |  |  | in this model | 0.92 (0.67-1.27) |
| Q3 |  | 1.14 (0.84-1.56) |  |  |  | 1.21 (0.87-1.68) |
| Q4 (highest) |  | 1.61 (1.18-2.19) |  |  |  | 1.67 (1.21-2.31) |
|  |  |  |  |  |  |  |
|  |  |  |  |  |  |  |
|  |  |  |  |  |  |  |
| mGPS |  |  |  |  |  |  |
| 0 | 1 (referent) | Did not remain |  |  | 1 (referent) | Did not remain |
| 1 | 2.09 (1.27-3.44) | in this model |  |  | 1.94 (1.17-3.24) | in this model |
| 2 | 1.63 (1.14-2.34) |  |  |  | 1.42 (1.00-2.03) |  |
|  |  |  |  |  |  |  |
| NLR |  |  |  |  |  |  |
| Q1 (lowest) | Did not remain | 1 (referent) |  |  | Did not remain | 1 (referent) |
| Q2 | in this model | 1.10 (0.81-1.49) |  |  | in this model | 1.09 (0.79-1.51) |
| Q3 |  | 1.28 (0.93-1.77) |  |  |  | 1.19 (0.85-1.68) |
| Q4 (highest) |  | 1.85 (1.34-2.56) |  |  |  | 1.65 (1.17-2.32) |
|  |  |  |  |  |  |  |

^a^ The multivariable Cox regression model initially included age, sex, body mass index, diabetes mellitus, PS, primary tumor site, cancer status, receipt of treatment, CA19-9, mGPS, and NLR. A backward elimination with a threshold *P* of 0.05 was conducted to select variables for the final models.

^b^ Sarcopenia was defined according to the cut-off values for SMI proposed by Japan Society of Hepatology guidelines [6]: SMI < 42 cm^2^/m^2^ for males and SMI < 38 cm^2^/m^2^ for females.

Abbreviations: CA19-9, carbohydrate antigen 19-9; CI, confidence interval; ECOG, Eastern Cooperative Oncology Group; HR, hazard ratio; mGPS, modified Glasgow prognostic score; NLR, neutrophil-to-lymphocyte ratio; PS, performance status; Q1-4, quartiles 1-4; SMD, skeletal muscle density; SMI, skeletal muscle index

**Supplementary Table 3** Sarcopenia defined by SMI and intramuscular adipose tissue content (IMAC) in relation to survival among patients with advanced biliary tract cancer

|  | Progression-free survival | | | |  | Overall survival | | | |
| --- | --- | --- | --- | --- | --- | --- | --- | --- | --- |
|  | No. of  cases | No. of  events | Univariable  HR (95% CI) | Multivariable  HR^a^ (95% CI) |  | No. of  cases | No. of  events | Univariable  HR (95% CI) | Multivariable  HR^a^ (95% CI) |
| Sarcopenia^b^ |  |  |  |  |  |  |  |  |  |
| Absent | 246 | 160 | 1 (referent) | 1 (referent) |  | 312 | 283 | 1 (referent) | 1 (referent) |
| Present | 23 | 16 | 1.62 (0.97-2.73) | 1.80 (1.05-3.08) |  | 47 | 42 | 1.51 (1.09-2.10) | 1.24 (0.88-1.75) |
|  |  |  |  |  |  |  |  |  |  |
| *P* |  |  | 0.067 | 0.032 |  |  |  | 0.013 | 0.23 |
|  |  |  |  |  |  |  |  |  |  |

^a^ The multivariable Cox regression model initially included age, sex, body mass index, diabetes mellitus, performance status, primary tumor site, cancer status, receipt of treatment, carbohydrate antigen 19-9, modified Glasgow prognostic score, and neutrophil-to-lymphocyte ratio. A backward elimination with a threshold *P* of 0.05 was conducted to select variables for the final models.

^b^ Sarcopenia was defined according to the cut-off values for SMI proposed by Japan Society of Hepatology guidelines for SMI (< 42 cm^2^/m^2^ for males and < 38 cm^2^/m^2^ for females) [6] and the highest sex-specific quartile of IMAC.

Abbreviations: CI, confidence interval; HR, hazard ratio; IMAC, intramuscular adipose tissue content; SMI, skeletal muscle index

**Supplementary Table 4** SMD at baseline standardized for computed tomography machines in relation to survival among patients with advanced biliary tract cancer^a^

|  | Progression-free survival | | | |  | Overall survival | | | |
| --- | --- | --- | --- | --- | --- | --- | --- | --- | --- |
|  | No. of  cases | No. of  events | Univariable  HR (95% CI) | Multivariable  HR^b^ (95% CI) |  | No. of  cases | No. of  events | Univariable  HR (95% CI) | Multivariable  HR^b^ (95% CI) |
| Standard scores of SMD at baseline^c^ |  |  |  |  |  |  |  |  |  |
| Q4 (highest) | 68 | 49 | 1 (referent) | 1 (referent) |  | 78 | 69 | 1 (referent) | 1 (referent) |
| Q3 | 67 | 44 | 0.89 (0.59-1.35) | 1.03 (0.67-1.60) |  | 79 | 73 | 0.99 (0.71-1.38) | 0.97 (0.69-1.36) |
| Q2 | 63 | 42 | 1.06 (0.70-1.61) | 1.00 (0.65-1.54) |  | 78 | 70 | 1.04 (0.74-1.45) | 0.93 (0.66-1.31) |
| Q1 (lowest) | 47 | 31 | 1.25 (0.80-1.96) | 1.27 (0.78-2.06) |  | 79 | 71 | 1.79 (1.28-2.50) | 1.52 (1.07-2.16) |
|  |  |  |  |  |  |  |  |  |  |
| *P*_trend_^d^ |  |  | 0.090 | 0.040 |  |  |  | 0.002 | 0.007 |
|  |  |  |  |  |  |  |  |  |  |

^a^ Cases with machines used for < 30 cases were excluded for a robust statistical assessment.

^b^ The multivariable Cox regression model initially included age, sex, body mass index, diabetes mellitus, performance status, primary tumor site, cancer status, receipt of treatment, carbohydrate antigen 19-9, modified Glasgow prognostic score, and neutrophil-to-lymphocyte ratio. A backward elimination with a threshold *P* of 0.05 was conducted to select variables for the final models.

^c^ Standard scores of SMD was calculated as (a value of a given case minus a machine-specific mean) / a machine-specific standard deviation.

^d^ *P*_trend_ was calculated by entering standard scores of SMD at baseline (continuous) in the Cox regression model.

Abbreviations: CI, confidence interval; HR, hazard ratio; Q1-4, quartiles 1-4; SMD, skeletal muscle density

**Supplementary Table 5** Saropenia and SMD at baseline in relation to survival among patients with advanced biliary tract cancer, stratified by tumor sites^a^

|  | Progression-free survival | | | |  | Overall survival | | | |
| --- | --- | --- | --- | --- | --- | --- | --- | --- | --- |
|  | No. of  cases | No. of  events | Univariable  HR (95% CI) | Multivariable  HR^b^ (95% CI) |  | No. of  cases | No. of  events | Univariable  HR (95% CI) | Multivariable  HR^b^ (95% CI) |
| **Intrahepatic bile duct** |  |  |  |  |  |  |  |  |  |
| Sarcopenia^c^ at baseline |  |  |  |  |  |  |  |  |  |
| Absent | 57 | 38 | 1 (referent) | 1 (referent) |  | 64 | 54 | 1 (referent) | 1 (referent) |
| Present | 36 | 22 | 1.53 (0.90-2.61) | 2.01 (1.15-3.52) |  | 41 | 41 | 1.91 (1.27-2.87) | 1.75 (1.15-2.67) |
|  |  |  |  |  |  |  |  |  |  |
| **Extrahepatic bile duct** |  |  |  |  |  |  |  |  |  |
| Sarcopenia^c^ at baseline |  |  |  |  |  |  |  |  |  |
| Absent | 64 | 39 | 1 (referent) | 1 (referent) |  | 93 | 83 | 1 (referent) | 1 (referent) |
| Present | 56 | 36 | 1.36 (0.86-2.15) | 1.49 (0.93-2.37) |  | 85 | 76 | 1.08 (0.79-1.47) | 1.19 (0.87-1.64) |
|  |  |  |  |  |  |  |  |  |  |
| **Gallbladder** |  |  |  |  |  |  |  |  |  |
| Sarcopenia^c^ at baseline |  |  |  |  |  |  |  |  |  |
| Absent | 41 | 27 | 1 (referent) | 1 (referent) |  | 47 | 44 | 1 (referent) | 1 (referent) |
| Present | 23 | 17 | 0.85 (0.46-1.56) | 1.15 (0.58-2.30) |  | 35 | 32 | 1.25 (0.79-1.98) | 1.02 (0.64-1.63) |
|  |  |  |  |  |  |  |  |  |  |
| *P*_interaction_^d^ |  |  | 0.33 | 0.43 |  |  |  | 0.091 | 0.21 |
|  |  |  |  |  |  |  |  |  |  |
| **Intrahepatic bile duct** |  |  |  |  |  |  |  |  |  |
| SMD at baseline |  |  |  |  |  |  |  |  |  |
| Q4 (highest) | 19 | 14 | 1 (referent) | 1 (referent) |  | 21 | 18 | 1 (referent) | 1 (referent) |
| Q3 | 28 | 17 | 0.80 (0.39-1.63) | 0.76 (0.37-1.56) |  | 31 | 29 | 1.59 (0.87-2.88) | 0.76 (0.40-1.44) |
| Q2 | 23 | 13 | 1.77 (0.81-3.83) | 1.84 (0.85-3.99) |  | 25 | 23 | 2.09 (1.12-3.92) | 1.59 (0.83-3.04) |
| Q1 (lowest) | 20 | 15 | 2.44 (1.15-5.16) | 2.48 (1.15-5.33) |  | 25 | 23 | 3.26 (1.73-6.15) | 1.79 (0.93-3.45) |
|  |  |  |  |  |  |  |  |  |  |
| **Extrahepatic bile duct** |  |  |  |  |  |  |  |  |  |
| SMD at baseline |  |  |  |  |  |  |  |  |  |
| Q4 (highest) | 34 | 22 | 1 (referent) | 1 (referent) |  | 43 | 37 | 1 (referent) | 1 (referent) |
| Q3 | 32 | 17 | 0.93 (0.49-1.75) | 0.88 (0.46-1.66) |  | 41 | 37 | 1.14 (0.72-1.80) | 1.14 (0.70-1.85) |
| Q2 | 24 | 19 | 0.91 (0.49-1.68) | 0.81 (0.43-1.51) |  | 42 | 38 | 0.96 (0.61-1.51) | 0.52 (0.32-0.85) |
| Q1 (lowest) | 21 | 12 | 0.81 (0.40-1.65) | 0.73 (0.36-1.50) |  | 41 | 37 | 1.46 (0.93-2.31) | 1.07 (0.64-1.77) |
|  |  |  |  |  |  |  |  |  |  |
|  |  |  |  |  |  |  |  |  |  |
|  |  |  |  |  |  |  |  |  |  |
| **Gallbladder** |  |  |  |  |  |  |  |  |  |
| SMD at baseline |  |  |  |  |  |  |  |  |  |
| Q4 (highest) | 19 | 14 | 1 (referent) | 1 (referent) |  | 20 | 17 | 1 (referent) | 1 (referent) |
| Q3 | 15 | 10 | 2.17 (0.95-4.94) | 2.31 (1.01-5.27) |  | 16 | 15 | 1.55 (0.77-3.11) | 1.27 (0.62-2.59) |
| Q2 | 15 | 12 | 2.50 (1.15-5.47) | 2.40 (1.10-5.27) |  | 19 | 19 | 2.30 (1.19-4.45) | 1.60 (0.80-3.19) |
| Q1 (lowest) | 9 | 5 | 1.90 (0.68-5.31) | 2.13 (0.75-6.02) |  | 19 | 18 | 3.45 (1.77-6.72) | 1.43 (0.69-2.98) |
|  |  |  |  |  |  |  |  |  |  |
| *P*_interaction_^d^ |  |  | 0.11 | 0.038 |  |  |  | 0.006 | 0.003 |
|  |  |  |  |  |  |  |  |  |  |

^a^ Due to the small sample size, cases with ampullary cancer were excluded from the analysis.

^b^ The multivariable Cox regression model initially included age, sex, body mass index, diabetes mellitus, performance status, cancer status, receipt of treatment, carbohydrate antigen 19-9, modified Glasgow prognostic score, and neutrophil-to-lymphocyte ratio. A backward elimination with a threshold *P* of 0.05 was conducted to select variables for the final models.

^c^ Sarcopenia was defined according to the cut-off values for SMI proposed by Japan Society of Hepatology guidelines [6]: SMI < 42 cm^2^/m^2^ for males and SMI < 38 cm^2^/m^2^ for females.

^d^ *P*_interaction_ was calculated by entering main effect terms and a cross-product term of sarcopenia (or SMD [quartile-specific medians]) and the tumor site into the model and evaluating the likelihood ratio test.

Abbreviations: CI, confidence interval; HR, hazard ratio; Q1-4, quartiles 1-4; SMD, skeletal muscle density; SMI, skeletal muscle index

**Supplementary Table 6** Saropenia and SMD at baseline in relation to survival among patients with advanced biliary tract cancer, stratified by chemotherapy regimens

|  | Progression-free survival | | | |  | Overall survival | | | |
| --- | --- | --- | --- | --- | --- | --- | --- | --- | --- |
|  | No. of  cases | No. of  events | Univariable  HR (95% CI) | Multivariable  HR^a^ (95% CI) |  | No. of  cases | No. of  events | Univariable  HR (95% CI) | Multivariable  HR^a^ (95% CI) |
| **Combination therapy (Gem/CDDP or Gem/S-1)** |  |  |  |  |  |  |  |  |  |
| Sarcopenia^b^ at baseline |  |  |  |  |  |  |  |  |  |
| Absent | 108 | 68 | 1 (referent) | 1 (referent) |  | 108 | 95 | 1 (referent) | 1 (referent) |
| Present | 82 | 56 | 1.26 (0.89-1.80) | 1.47 (0.99-2.18) |  | 82 | 76 | 1.23 (0.91-1.67) | 1.24 (0.92-1.69) |
|  |  |  |  |  |  |  |  |  |  |
| **Monotherapy (Gem or S-1)** |  |  |  |  |  |  |  |  |  |
| Sarcopenia^b^ at baseline |  |  |  |  |  |  |  |  |  |
| Absent | 47 | 29 | 1 (referent) | 1 (referent) |  | 47 | 44 | 1 (referent) | 1 (referent) |
| Present | 39 | 23 | 1.23 (0.71-2.13) | 1.82 (1.02-3.28) |  | 39 | 36 | 1.13 (0.73-1.76) | 1.25 (0.80-1.97) |
|  |  |  |  |  |  |  |  |  |  |
| **Best supportive care^c^** |  |  |  |  |  |  |  |  |  |
| Sarcopenia^b^ at baseline |  |  |  |  |  |  |  |  |  |
| Absent | - | - | - | - |  | 39 | 36 | 1 (referent) | 1 (referent) |
| Present | - | - | - | - |  | 43 | 39 | 1.08 (0.68-1.71) | 1.12 (0.70-1.78) |
|  |  |  |  |  |  |  |  |  |  |
| *P*_interaction_^d^ |  |  | 0.93 | 0.53 |  |  |  | 0.88 | 0.92 |
|  |  |  |  |  |  |  |  |  |  |
| **Combination therapy (Gem/CDDP or Gem/S-1)** |  |  |  |  |  |  |  |  |  |
| SMD at baseline |  |  |  |  |  |  |  |  |  |
| Q4 (highest) | 54 | 34 | 1 (referent) | 1 (referent) |  | 54 | 48 | 1 (referent) | 1 (referent) |
| Q3 | 50 | 34 | 1.29 (0.80-2.08) | 1.40 (0.86-2.28) |  | 50 | 46 | 1.29 (0.86-1.93) | 1.08 (0.71-1.65) |
| Q2 | 46 | 31 | 1.33 (0.82-2.17) | 1.31 (0.79-2.16) |  | 46 | 44 | 1.20 (0.80-1.81) | 1.06 (0.68-1.63) |
| Q1 (lowest) | 30 | 18 | 1.24 (0.70-2.20) | 1.42 (0.77-2.59) |  | 30 | 24 | 1.20 (0.73-1.96) | 1.10 (0.67-1.82) |
|  |  |  |  |  |  |  |  |  |  |
| **Monotherapy (Gem or S-1)** |  |  |  |  |  |  |  |  |  |
| SMD at baseline |  |  |  |  |  |  |  |  |  |
| Q4 (highest) | 12 | 10 | 1 (referent) | 1 (referent) |  | 12 | 11 | 1 (referent) | 1 (referent) |
| Q3 | 26 | 11 | 0.42 (0.18-0.99) | 0.46 (0.19-1.16) |  | 26 | 24 | 0.78 (0.38-1.60) | 0.86 (0.40-1.83) |
| Q2 | 16 | 12 | 1.06 (0.46-2.45) | 1.73 (0.70-4.24) |  | 16 | 14 | 1.30 (0.59-2.87) | 1.62 (0.70-3.76) |
| Q1 (lowest) | 20 | 13 | 1.27 (0.56-2.91) | 1.59 (0.68-3.71) |  | 20 | 20 | 1.84 (0.88-3.86) | 2.16 (1.00-4.69) |
|  |  |  |  |  |  |  |  |  |  |
|  |  |  |  |  |  |  |  |  |  |
|  |  |  |  |  |  |  |  |  |  |
| **Best supportive care^c^** |  |  |  |  |  |  |  |  |  |
| SMD at baseline |  |  |  |  |  |  |  |  |  |
| Q4 (highest) | - | - | - | - |  | 8 | 6 | 1 (referent) | 1 (referent) |
| Q3 | - | - | - | - |  | 10 | 10 | 3.95 (1.43-10.93) | 3.33 (1.17-9.42) |
| Q2 | - | - | - | - |  | 23 | 22 | 1.35 (0.54-3.34) | 0.93 (0.37-2.32) |
| Q1 (lowest) | - | - | - | - |  | 36 | 34 | 3.06 (1.28-7.30) | 2.05 (0.83-5.03) |
|  |  |  |  |  |  |  |  |  |  |
| *P*_interaction_^d^ |  |  | 0.60 | 0.44 |  |  |  | 0.49 | 0.16 |
|  |  |  |  |  |  |  |  |  |  |

^a^ The multivariable Cox regression model initially included age, sex, body mass index, diabetes mellitus, performance status, cancer status, receipt of treatment, carbohydrate antigen 19-9, modified Glasgow prognostic score, and neutrophil-to-lymphocyte ratio. A backward elimination with a threshold *P* of 0.05 was conducted to select variables for the final models.

^b^ Sarcopenia was defined according to the cut-off values for SMI proposed by Japan Society of Hepatology guidelines [6]: SMI < 42 cm^2^/m^2^ for males and SMI < 38 cm^2^/m^2^ for females.

^c^ Cases with best supportive care were excluded from the analyses of PFS.

^d^ *P*_interaction_ was calculated by entering main effect terms and a cross-product term of sarcopenia (or SMD [quartile-specific medians]) and the chemotherapy regimen into the model and evaluating the likelihood ratio test.

Abbreviations: CDDP, cisplatin; CI, confidence interval; Gem, gemcitabine; HR, hazard ratio; Q1-4, quartiles 1-4; SMD, skeletal muscle density; SMI, skeletal muscle index

**Supplementary Table 7** SMI at baseline in relation to survival among patients with advanced biliary tract cancer

|  | Progression-free survival | | | |  | Overall survival | | | |
| --- | --- | --- | --- | --- | --- | --- | --- | --- | --- |
|  | No. of  cases | No. of  events | Univariable  HR (95% CI) | Multivariable  HR^a^ (95% CI) |  | No. of  cases | No. of  events | Univariable  HR (95% CI) | Multivariable  HR^a^ (95% CI) |
| SMI at baseline^b^ |  |  |  |  |  |  |  |  |  |
| Q4 (highest) | 75 | 42 | 1 (referent) | 1 (referent) |  | 96 | 84 | 1 (referent) | 1 (referent) |
| Q3 | 77 | 55 | 1.03 (0.69-1.54) | 1.09 (0.71-1.68) |  | 97 | 88 | 0.91 (0.68-1.23) | 0.95 (0.70-1.29) |
| Q2 | 71 | 48 | 0.83 (0.55-1.25) | 0.88 (0.55-1.40) |  | 96 | 85 | 0.79 (0.59-1.07) | 0.84 (0.62-1.15) |
| Q1 (lowest) | 68 | 44 | 1.36 (0.89-2.08) | 1.86 (1.12-3.09) |  | 97 | 91 | 1.37 (1.02-1.85) | 1.40 (1.03-1.91) |
|  |  |  |  |  |  |  |  |  |  |
| *P*_trend_^c^ |  |  | 0.40 | 0.15 |  |  |  | 0.29 | 0.59 |
|  |  |  |  |  |  |  |  |  |  |

^a^ The multivariable Cox regression model initially included age, sex, body mass index, diabetes mellitus, performance status, primary tumor site, cancer status, receipt of treatment, carbohydrate antigen 19-9, modified Glasgow prognostic score, and neutrophil-to-lymphocyte ratio. A backward elimination with a threshold *P* of 0.05 was conducted to select variables for the final models.

^b^ The SMI at baseline was categorized into Q1 (28.3-40.3 cm^2^/m^2^), Q2 (40.4-44.7 cm^2^/m^2^), Q3 (44.8-49.9 cm^2^/m^2^), and Q4 (50.0-67.4 cm^2^/m^2^) for males and Q1 (20.7-31.8 cm^2^/m^2^), Q2 (31.9-35.9 cm^2^/m^2^), Q3 (36.0-40.5 cm^2^/m^2^), and Q4 (40.6-53.9 cm^2^/m^2^) for females.

^c^ *P*_trend_ was calculated by entering quartile-specific median values of SMI at baseline (continuous) in the Cox regression model.

Abbreviations: CI, confidence interval; HR, hazard ratio; Q1-4, quartiles 1-4; SMI, skeletal muscle index

**Supplementary Table 8**  SMI and SMD change in 2-4 months in relation to survival among patients with advanced biliary tract cancer (the final multivariable models)

|  | Multivariable HR^a^ (95% CI) | |  |  | Multivariable HR^a^ (95% CI) | |
| --- | --- | --- | --- | --- | --- | --- |
|  | Progression-free survival | Overall survival |  |  | Progression-free survival | Overall survival |
| SMI change in 2-4 months |  |  |  | SMD change in 2-4 months |  |  |
| Q4 (less loss or gain) | 1 (referent) | 1 (referent) |  | Q4 (less loss or gain) | 1 (referent) | 1 (referent) |
| Q3 | 0.96 (0.63-1.46) | 0.85 (0.60-1.21) |  | Q3 | 1.25 (0.77-2.00) | 1.10 (0.74-1.64) |
| Q2 | 0.97 (0.65-1.45) | 0.78 (0.54-1.13) |  | Q2 | 1.36 (0.84-2.20) | 0.86 (0.58-1.29) |
| Q1 (more loss) | 0.95 (0.60-1.50) | 1.66 (1.17-2.36) |  | Q1 (more loss) | 1.82 (1.13-2.94) | 1.17 (0.78-1.74) |
|  |  |  |  |  |  |  |
| Age | Did not remain | 1 (referent) |  |  | Did not remain | 1 (referent) |
| (per 10-year increase) | in this model | 1.14 (1.00-1.31) |  |  | in this model | 1.17 (1.01-1.35) |
|  |  |  |  |  |  |  |
| BMI, kg/m^2^ |  |  |  |  |  |  |
| 18.5-25.0 | Did not remain | Did not remain |  |  | 1 (referent) | Did not remain |
| <18.5 | in this model | in this model |  |  | 0.71 (0.41-1.22) | in this model |
| >25.0 |  |  |  |  | 1.68 (1.06-2.64) |  |
|  |  |  |  |  |  |  |
| Primary tumor site |  |  |  |  |  |  |
| Intrahepatic | 1 (referent) | 1 (referent) |  |  | 1 (referent) | 1 (referent) |
| Extrahepatic | 0.96 (0.67-1.38) | 1.12 (0.82-1.53) |  |  | 1.24 (0.79-1.93) | 0.95 (0.67-1.34) |
| Gallbladder | 1.85 (1.23-2.78) | 1.79 (1.24-2.58) |  |  | 2.31 (1.44-3.70) | 1.67 (1.11-2.51) |
| Ampulla | 1.76 (0.85-3.65) | 1.06 (0.55-2.05) |  |  | 1.69 (0.68-4.20) | 0.82 (0.38-1.75) |
|  |  |  |  |  |  |  |
| Cancer status |  |  |  |  |  |  |
| Localized | Did not remain | Did not remain |  |  | 1 (referent) | Did not remain |
| Metastatic | in this model | in this model |  |  | 1.48 (1.03-2.11) | in this model |
|  |  |  |  |  |  |  |
| Treatment |  |  |  |  |  |  |
| Combination chemotherapy | Did not remain | 1 (referent) |  |  | Did not remain | 1 (referent) |
| Single agent chemotherapy | in this model | 1.11 (0.82-1.50) |  |  | in this model | 1.02 (0.73-1.41) |
| Radiation |  | 0.50 (0.24-1.05) |  |  |  | 0.35 (0.14-0.88) |
| Best supportive care |  | 1.95 (1.23-3.11) |  |  |  | 2.11 (1.27-3.49) |
|  |  |  |  |  |  |  |
|  |  |  |  |  |  |  |
|  |  |  |  |  |  |  |
|  |  |  |  |  |  |  |
|  |  |  |  |  |  |  |
| CA19-9 |  |  |  |  |  |  |
| Q1 (lowest) | Did not remain | 1 (referent) |  |  | Did not remain | 1 (referent) |
| Q2 | in this model | 0.88 (0.61-1.26) |  |  | in this model | 0.89 (0.58-1.35) |
| Q3 |  | 1.04 (0.73-1.47) |  |  |  | 1.25 (0.84-1.85) |
| Q4 (highest) |  | 1.76 (1.25-2.49) |  |  |  | 1.73 (1.18-2.52) |
|  |  |  |  |  |  |  |
| mGPS |  |  |  |  |  |  |
| 0 | 1 (referent) | Did not remain |  |  | 1 (referent) | Did not remain |
| 1 | 2.10 (1.25-3.53) | in this model |  |  | 2.21 (1.28-3.80) | in this model |
| 2 | 1.54 (1.08-2.19) |  |  |  | 1.60 (1.07-2.39) |  |
|  |  |  |  |  |  |  |
| NLR |  |  |  |  |  |  |
| Q1 (lowest) | Did not remain | 1 (referent) |  |  | Did not remain | 1 (referent) |
| Q2 | in this model | 1.16 (0.83-1.62) |  |  | in this model | 1.11 (0.77-1.62) |
| Q3 |  | 1.34 (0.94-1.92) |  |  |  | 1.29 (0.86-1.94) |
| Q4 (highest) |  | 1.91 (1.31-2.78) |  |  |  | 1.84 (1.22-2.79) |
|  |  |  |  |  |  |  |

^a^ The multivariable Cox regression model initially included age, sex, BMI, diabetes mellitus, performance status, primary tumor site, cancer status, receipt of treatment, CA19-9, mGPS, and NLR. A backward elimination with a threshold *P* of 0.05 was conducted to select variables for the final models.

Abbreviations: BMI, body mass index; CA19-9, carbohydrate antigen 19-9; CI, confidence interval; HR, hazard ratio; mGPS, modified Glasgow prognostic score; NLR, neutrophil-to-lymphocyte ratio; Q1-4, quartiles 1-4; SMD, skeletal muscle density; SMI, skeletal muscle index

**Supplementary Table 9** SMI and SMD changes in 2-4 months in relation to survival among patients with advanced biliary tract cancer, stratified by the baseline status

|  | Progression-free survival | | | |  | Overall survival | | | |
| --- | --- | --- | --- | --- | --- | --- | --- | --- | --- |
|  | No. of  cases | No. of  events | Univariable  HR (95% CI) | Multivariable  HR^a^ (95% CI) |  | No. of  cases | No. of  events | Univariable  HR (95% CI) | Multivariable  HR^a^ (95% CI) |
| **No sarcopenia^b^ at baseline** |  |  |  |  |  |  |  |  |  |
| Decrease in SMI (per 10% decrease) | 148 | 104 | 1.10 (0.82-1.47) | 1.22 (0.92-1.62) |  | 169 | 149 | 1.37 (1.07-1.76) | 1.42 (1.11-1.81) |
|  |  |  |  |  |  |  |  |  |  |
| **Sarcopenia^b^ at baseline** |  |  |  |  |  |  |  |  |  |
| Decrease in SMI (per 10% decrease) | 107 | 79 | 1.06 (0.79-1.42) | 0.95 (0.70-1.30) |  | 128 | 117 | 1.35 (1.06-1.71) | 1.27 (1.00-1.59) |
|  |  |  |  |  |  |  |  |  |  |
| *P*_interaction_^c^ |  |  | 0.86 | 0.25 |  |  |  | 0.91 | 0.50 |
|  |  |  |  |  |  |  |  |  |  |
| **SMD at baseline, Q4 (highest)** |  |  |  |  |  |  |  |  |  |
| Decrease in SMD (per 10% decrease) | 62 | 45 | 1.50 (1.08-2.07) | 1.28 (0.87-1.88) |  | 70 | 60 | 1.11 (0.82-1.50) | 1.10 (0.81-1.51) |
|  |  |  |  |  |  |  |  |  |  |
| **SMD at baseline, Q3** |  |  |  |  |  |  |  |  |  |
| Decrease in SMD (per 10% decrease) | 57 | 39 | 1.09 (0.86-1.38) | 1.30 (1.03-1.65) |  | 65 | 59 | 1.14 (0.92-1.42) | 1.21 (1.00-1.47) |
|  |  |  |  |  |  |  |  |  |  |
| **SMD at baseline, Q2** |  |  |  |  |  |  |  |  |  |
| Decrease in SMD (per 10% decrease) | 53 | 40 | 1.27 (1.03-1.57) | 1.15 (0.93-1.42) |  | 64 | 58 | 1.19 (0.99-1.43) | 1.11 (0.92-1.34) |
|  |  |  |  |  |  |  |  |  |  |
| **SMD at baseline, Q1 (lowest)** |  |  |  |  |  |  |  |  |  |
| Decrease in SMD (per 10% decrease) | 42 | 29 | 1.15 (0.92-1.44) | 1.27 (1.01-1.60) |  | 49 | 44 | 1.06 (0.88-1.28) | 1.10 (0.93-1.31) |
|  |  |  |  |  |  |  |  |  |  |
| *P*_interaction_^c^ |  |  | 0.44 | 0.57 |  |  |  | 0.91 | 0.80 |
|  |  |  |  |  |  |  |  |  |  |

^a^ The multivariable Cox regression model initially included age, sex, body mass index, diabetes mellitus, performance status, cancer status, receipt of treatment, carbohydrate antigen 19-9, modified Glasgow prognostic score, and neutrophil-to-lymphocyte ratio. A backward elimination with a threshold *P* of 0.05 was conducted to select variables for the final models.

^b^ Sarcopenia was defined according to the cut-off values for SMI proposed by Japan Society of Hepatology guidelines [6]: SMI < 42 cm^2^/m^2^ for males and SMI < 38 cm^2^/m^2^ for females.

^c^ *P*_interaction_ was calculated by entering main effect terms and a cross-product term of a decrease in SMI (or SMD, percent, continuous) and the baseline status (sarcopenia or SMD [quartile-specific medians]) into the model and evaluating the likelihood ratio test.

Abbreviations: CI, confidence interval; HR, hazard ratio; Q1-4, quartiles 1-4; SMD, skeletal muscle density; SMI, skeletal muscle index

**Supplementary Table 10** Sarcopenia at baseline in relation to cholangitis in 2-4 months among patients with advanced biliary tract cancer

|  | Cholangitis in 2-4 months | | | |
| --- | --- | --- | --- | --- |
|  | No. of  cases | No. of  events | Univariable  OR (95% CI) | Multivariable  OR^a^ (95% CI) |
| **Sarcopenia^b^ at baseline** |  |  |  |  |
| Absent | 169 | 36 | 1 (referent) | 1 (referent) |
| Present | 128 | 40 | 1.68 (0.99-2.84) | 1.41 (0.78-2.56) |
|  |  |  |  |  |
| *P* |  |  | 0.053 | 0.25 |
|  |  |  |  |  |

^a^ The multivariable logistic regression model initially included age, sex, body mass index, diabetes mellitus, performance status, cancer status, receipt of treatment, carbohydrate antigen 19-9, modified Glasgow prognostic score, and neutrophil-to-lymphocyte ratio. A backward elimination with a threshold *P* of 0.05 was conducted to select variables for the final models.

^b^ Sarcopenia was defined according to the cut-off values for SMI proposed by Japan Society of Hepatology guidelines [6]: SMI < 42 cm^2^/m^2^ for males and SMI < 38 cm^2^/m^2^ for females.

Abbreviations: CI, confidence interval; OR, odds ratio; SMI, skeletal muscle index

**
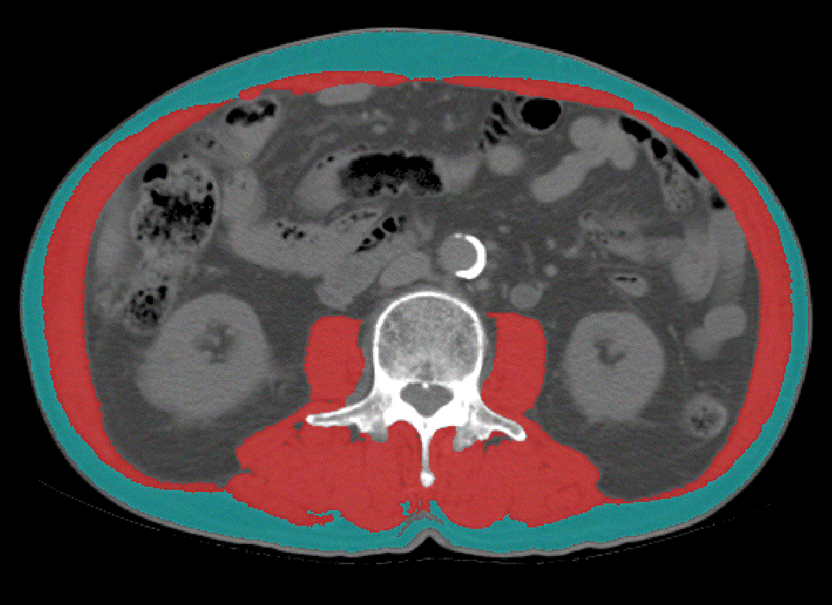
**

**Supplementary Fig. 1** Computed tomography-based assessment of intramuscular adipose tissue content at the level of the third lumbar vertebra. The intramuscular adipose tissue content was calculated as the mean HU measurement of all skeletal muscles (red) divided by that of subcutaneous fat (blue).

HU, Hounsfield unit


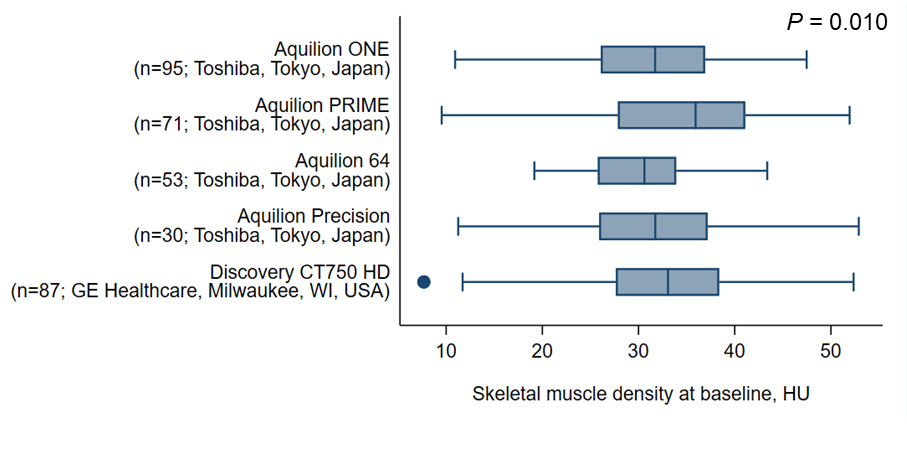


**Supplementary Fig. 2** Box plot of skeletal muscle densities by computed tomography machines among patients with advanced biliary tract cancer. *P* value was calculated by the Kruskal-Wallis test. Machines used for < 30 cases were excluded for a robust statistical assessment.

HU, Hounsfield unit

**
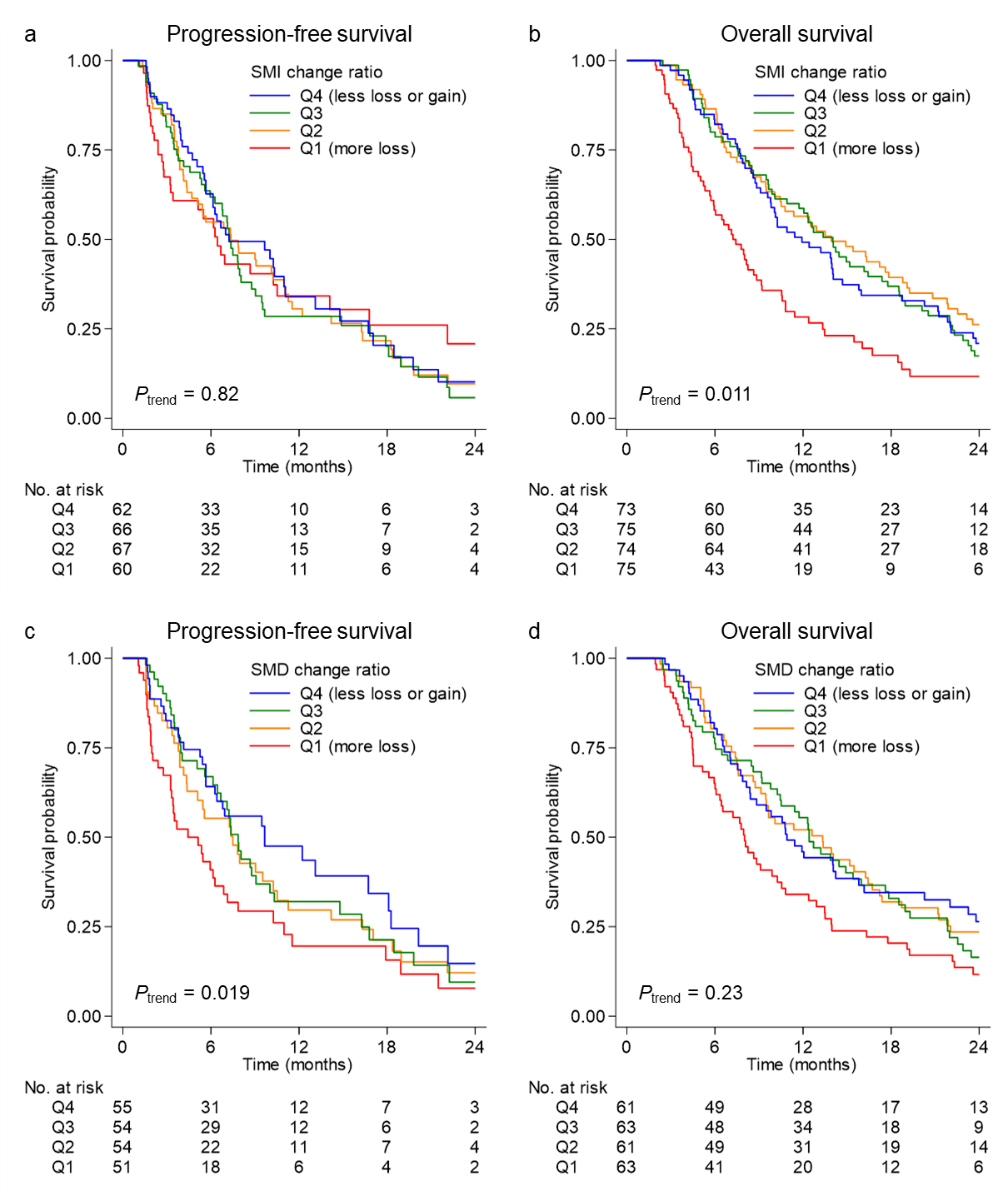
**

**Supplementary Fig. 3** Kaplan-Meier survival curves of patients with advanced biliary tract cancer according to serial changes of skeletal muscle status. a and b. PFS and OS, respectively, by SMI change in 2-4 months. c and d. PFS and OS, respectively, by SMD change in 2-4 months. For the lowest to highest quartiles of SMI change in 2-4 months, the median PFS times were 6.4 (95% CI, 3.3-10.5), 7.4 (95% CI, 4.7-11.0), 7.3 (95% CI, 5.9-8.8), and 7.2 months (95% CI, 5.7-11.1 months), respectively, and the median OS times were 7.2 (95% CI, 5.7-8.8), 13.9 (95% CI, 10.1-18.8), 14.0 (95% CI, 10.1-17.4), and 11.9 months (95% CI, 9.6-14.1 months), respectively. For the lowest to highest quartiles of SMD change in 2-4 months, the median PFS times were 4.4 (95% CI, 3.3-6.3), 7.5 (95% CI, 4.3-10.3), 7.9 (95% CI, 6.1-10.0), and 9.7 months (95% CI, 5.7-18.1 months), respectively, and the median OS times were 8.0 (95% CI, 6.1-10.3), 13.3 (95% CI, 9.1-16.4), 12.4 (95% CI, 10.3-16.0), and 10.9 months (95% CI, 8.4-15.8 months), respectively.

CI, confidence interval; OS, overall survival; PFS, progression-free survival; SMD, skeletal muscle density; SMI, skeletal muscle index

**
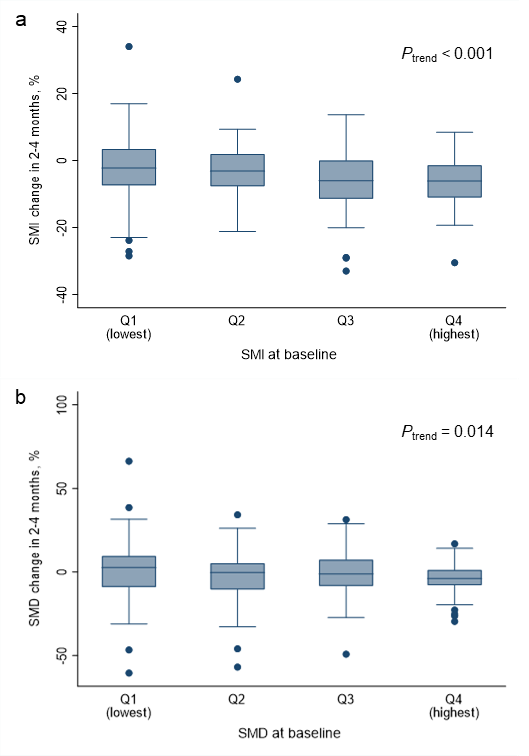
**

**Supplementary Fig. 4** Box plots of SMI and SMD changes in 2-4 months according to the corresponding metrics at baseline among patients with advanced biliary tract cancer. a. SMI. b. SMD. *P*_trend_ was calculated by the Jonckheere-Terpstra trend test.

SMD, skeletal muscle density; SMI, skeletal muscle index

**References**

1. Dolan RD, McSorley ST, Horgan PG, et al (2017) The role of the systemic inflammatory response in predicting outcomes in patients with advanced inoperable cancer: Systematic review and meta-analysis. Crit Rev Oncol Hematol 116:134-46

2. McMillan DC, Crozier JE, Canna K, et al (2007) Evaluation of an inflammation-based prognostic score (GPS) in patients undergoing resection for colon and rectal cancer. Int J Colorectal Dis 22:881-6

3. Hamada T, Cao Y, Qian ZR, et al (2017) Aspirin Use and Colorectal Cancer Survival According to Tumor CD274 (Programmed Cell Death 1 Ligand 1) Expression Status. J Clin Oncol 35:1836-44

4. Kiriyama S, Kozaka K, Takada T, et al (2018) Tokyo Guidelines 2018: diagnostic criteria and severity grading of acute cholangitis (with videos). J Hepatobiliary Pancreat Sci 25:17-30

5. Takahara N, Nakai Y, Isayama H, et al (2023) A prospective multicenter phase II study of FOLFIRINOX as a first-line treatment for patients with advanced and recurrent biliary tract cancer. Invest New Drugs 41:76-85

6. Nishikawa H, Shiraki M, Hiramatsu A, et al (2016) Japan Society of Hepatology guidelines for sarcopenia in liver disease (1st edition): Recommendation from the working group for creation of sarcopenia assessment criteria. Hepatol Res 46:951-63
